# Creating Cell‐Based Hybrid Noodles for Sustainable and Nutrient‐Balanced Diets via a Serum‐Free and Animal‐Free 3D Co‐Differentiation System

**DOI:** 10.1002/advs.202519916

**Published:** 2026-01-12

**Authors:** Xin Guan, Luyi Wang, Wanqiang Sun, Yiwei Feng, Dandan Wang, Haohao Tang, Zhenwu Ma, Guocheng Du, Jian Chen, Jingwen Zhou

**Affiliations:** ^1^ School of Biotechnology and Key Laboratory of Industrial Biotechnology of Ministry of Education Jiangnan University Wuxi China; ^2^ State Key Laboratory of Food Science and Resources Jiangnan University Wuxi China; ^3^ College of Mechanical Engineering Suzhou University of Science and Technology Suzhou China

**Keywords:** animal‐free scaffolds, cell‐based foods, co‐culture, co‐differentiation, hybrid foods, serum‐free

## Abstract

The growing demand for sustainable and nutritionally balanced food has fueled innovation in cell‐based hybrid foods. Here, we introduce an approach for developing hybrid noodles that combine carbohydrates, proteins, and fats through the 3D co‐culture and co‐differentiation of porcine muscle stem cells (pMuSCs) and mesenchymal stem cells (pMSCs) on engineered animal‐free scaffolds. We demonstrate that co‐culture with pMSCs significantly enhances the myogenic efficiency of late‐passage pMuSCs and reveal key signaling pathways mediating this effect. Importantly, we establish a streamlined co‐differentiation system that achieves simultaneous generation of myotubes and adipocytes in 3 days, without relying on serum or conventional chemical inducers. Thereafter, we develop an edible, starch‐based scaffold with excellent mechanical strength and cytocompatibility, leading to the successful production of cell‐based hybrid noodles. This hybrid noodle exhibits a balanced macronutrient profile, enhanced texture, and a distinct meaty flavor compared to conventional starch‐based noodles. This study offers a promising approach to establishing sustainable and nutritionally complete food systems.

## Graphical abstract

This study develops a 3D co‐culture and co‐differentiation system for porcine muscle stem cells (pMuSCs) and mesenchymal stem cells (pMSCs) on edible starch‐based scaffolds. The system simultaneously generates myotubes and adipocytes without using serum or chemical inducers. The resulting cell‐based hybrid noodles combine balanced nutrition, improved texture, and meaty flavor.

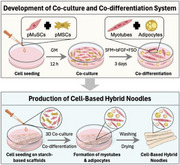

## 1 Introduction

As the global population grows and climate change intensifies, creating sustainable food systems has become crucial. This has led to the development of various eco‐friendly and healthy food sources, such as plant, microbial, insect, and microalgae proteins and cell‐based foods [1]. Concurrently, growing consumer demand for diverse and innovative food products has driven the market to offer a wide range of novel food forms. Among them, hybrid foods, which integrate ingredients from various sources such as grains, vegetables, and meats, have become a significant branch [2]. These foods offer balanced nutrition, good taste, and innovative eating experiences [3]. Notably, the advent of cell‐based food technology has provided a broader and more promising platform for the sustainable and diversified production of hybrid foods. By culturing different animal cells on substrates like plant‐derived proteins, vegetable leaves, and rice grains, a wide variety of cell‐based hybrid foods can be made [4, 5, 6]. In particular, their taste, nutrition, and flavor can be flexibly adjusted by changing the cells, substrates, medium formula, or cell‐to‐substrate ratio, at a significantly lower cost than cell‐based meat, in which cell biomass is the main ingredient of the product. Thus, cell‐based hybrid foods offer new strategies for achieving more sustainable, nutritionally balanced, and cost‐effective diets.

A healthy diet for adults typically derives 50 %–65 % of calories from carbohydrates, 10 %–15 % from proteins, and 20 %–35 % from fats [7]. Noodles, an excellent source of carbohydrates, play a vital role in the daily diets of many countries and regions worldwide. From a hybrid food perspective, incorporating protein and fat into noodles holds promise for creating enhanced food products with superior nutritional profiles and flavor. Of the two noodle ingredients, wheat flour and starch, starch noodles exhibit a more translucent appearance and a softer, more delicate texture. This is attributed to the gelatinization of starch granules upon heating, which facilitates the formation of a homogeneous gel with greater mechanical strength [8]. Also encouragingly, starch exhibits good biocompatibility and low immunogenicity, making it suitable for development as a biomaterial for use in fields such as tissue engineering, controlled release of drugs, and medical implants [9]. It has been demonstrated that starch can be combined with gellan gum, gelatin, or magnesium–calcium pyrophosphate to fabricate cell scaffolds, which are effective in supporting the attachment, proliferation, and function of different types of cells, such as bone marrow stromal cells, schwann cells, mouse embryo osteoblast precursor cells (MC3T3‐E1), and human umbilical vein endothelial cells (HUVECs) [10, 11, 12, 13]. Therefore, we propose using starch as a cell scaffold to culture protein‐ and lipid‐rich animal cells, thereby transforming noodles into a nutrient‐rich hybrid food. This innovative approach will elevate noodles from a mere carbohydrate source into a hybrid food that offers superior nutrition, flavor, and texture, and also aligns with the pursuit of sustainable, healthy, and diversified diets.

Myotubes and adipocytes are the primary cellular sources of animal protein and fat, respectively [14, 15]. Recent advances in cell‐based meat technology have enabled the in vitro production of myotubes and adipocytes under 2D or 3D culture conditions. For instance, Zhu et al. demonstrated the successful generation of myotubes from porcine pre‐gastrulation epiblast stem cells (pgEpiSCs) with an edible konjac glucomannan (KGM)‐sodium alginate (SA) scaffold [16]. Zhou et al. fabricated cultured fish fillets containing myotubes and adipocytes by proliferating and differentiating muscle satellite cells (MuSCs) and adipose‐derived stem cells (ASCs) from large yellow croaker on macroporous gelatin‐based microcarrier scaffolds [17]. However, existing approaches typically culture muscle and fat cells separately before assembly [18], resulting in stratified products that lack the integrated texture and sensory qualities in naturally interwoven tissues. Although recent studies have reported co‐production systems for myotubes and adipocytes [19, 20], these approaches remain critically dependent on animal serum and non‐food‐grade chemical inducers to ensure robust cell viability and lineage‐specific differentiation. Therefore, developing an efficient, serum‐free, and food‐safe co‐culture system that simultaneously generates myotubes and adipocytes is crucial for creating cell‐based hybrid foods with a homogeneous texture and desirable nutritional balance.

MuSCs are adult stem cells that reside in the muscle tissue, representing the predominant cell source for producing myotubes due to their potential for in vitro proliferation and straightforward myogenic differentiation. However, it is well‐documented that MuSCs suffer a severe loss of myogenic potential with prolonged culture time, resulting in a significant reduction in both the number and fusion index of generated myofibers [21]. We here developed a co‐culture system of porcine MuSCs (pMuSCs) and porcine mesenchymal stem cells (pMSCs), which not only improved the myogenic efficiency of long‐term expanded pMuSCs but also achieved the simultaneous generation of myotubes and adipocytes using a carefully designed serum‐free, chemical‐inducer‐free, rapid co‐differentiation medium. Thereafter, we fabricated starch‐based scaffolds and created cell‐based hybrid noodles by co‐culturing and co‐differentiating pMuSCs and pMSCs. The key nutritional characteristics and flavor profiles of the hybrid noodles were also evaluated.

## 2 Results

### 2.1 Co‐Culture With pMSCs Promotes Myotube Production of pMuSCs

**FIGURE 1:**
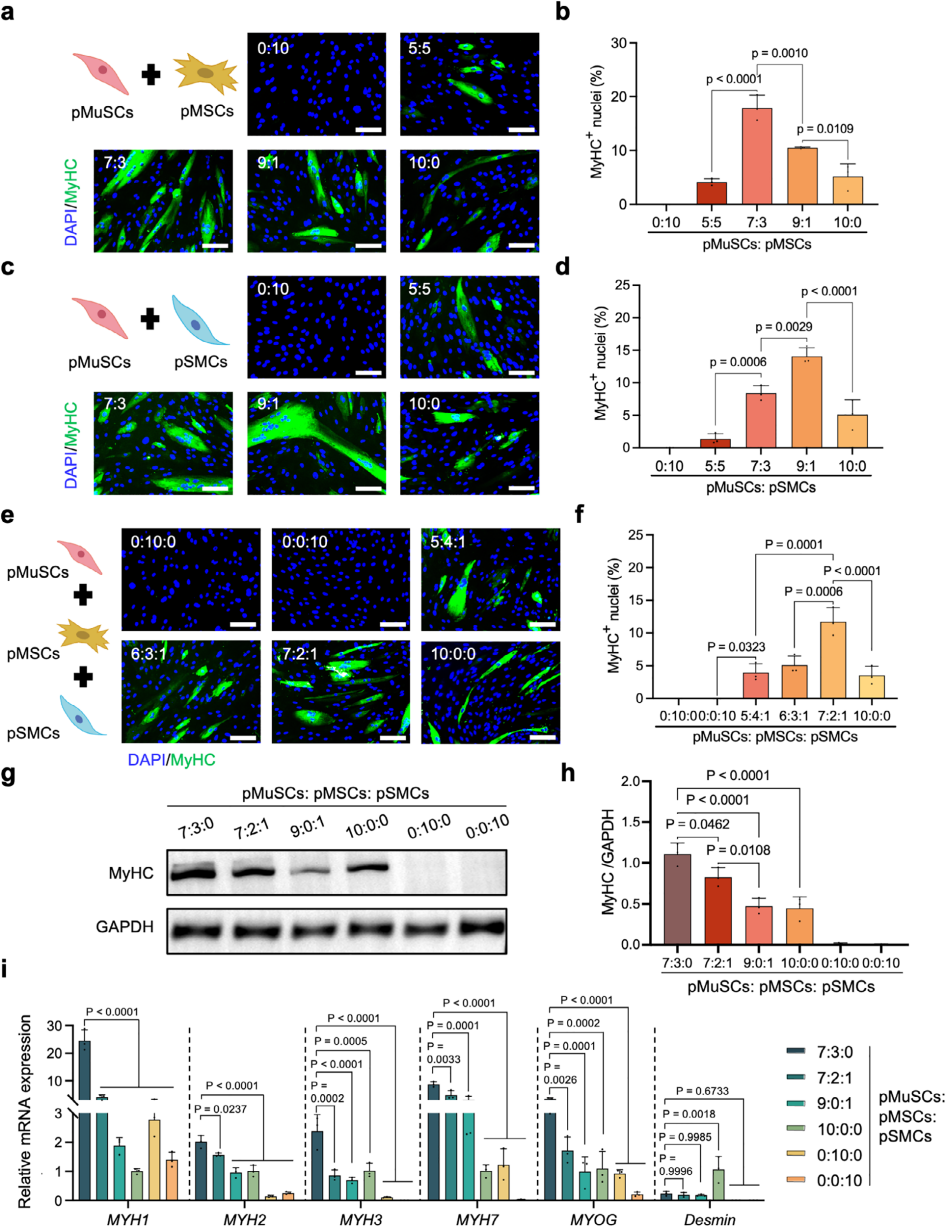
Co‐culture promotes myotube production of pMuSCs. (a) Representative MyHC immunofluorescence staining images after co‐culturing pMuSCs and pMSCs at different ratios for 3 days. Scale bar: 100 µm. (b) Quantification of the percentage of MyHC^+^ nuclei after co‐culturing pMuSCs and pMSCs at different ratios for 3 days (n = 3 independent experiments). (c) Representative MyHC immunofluorescence staining images after co‐culturing pMuSCs and pSMCs at different ratios for 3 days. Scale bar: 100 µm. (d) Quantification of the percentage of MyHC^+^ nuclei after co‐culturing pMuSCs and pSMCs at different ratios for 3 days (n = 3 independent experiments). (e) Representative MyHC immunofluorescence staining images after co‐culturing pMuSCs, pMSCs, and pSMCs at different ratios for 3 days. Scale bar: 100 µm. (f) Quantification of the percentage of MyHC^+^ nuclei after co‐culturing pMuSCs, pMSCs, and pSMCs at different ratios for 3 days (n = 3 independent experiments). (g) Representative blots of MyHC and GAPDH proteins after co‐culturing pMuSCs, pMSCs, and pSMCs at different combinations and ratios for 3 days. (h) Quantitation of MyHC protein expression normalized to GAPDH (n = 3 independent experiments). (i) The relative mRNA expression of *MYH1*, *MYH2*, *MYH3*, *MYH7*, *MYOG*, and *Desmin* genes after co‐culturing pMuSCs, pMSCs, and pSMCs at different combinations and ratios for 3 days (n = 3 independent experiments). For (b, d, f, h, i), error bars indicate means ± SD. Significance was determined by one‐way ANOVA with Tukey's post hoc analysis. *p*‐values are annotated in the figures. For (a, c, e, g), similar results were obtained in three independent experiments.

First, the myogenic potential of pMuSCs was evaluated during long‐term in vitro expansion, showing a significant decline in the number of myosin heavy chain‐positive (MyHC^+^) myotubes generated by pMuSCs in 2 % horse serum (HS) from passage (P)4 to P12 (Figure S1a). Notably, more than 60 % of the P4 pMuSCs were capable of myogenic differentiation, which plummeted to less than 7 % by P12 (Figure S1b). Given that cell expansion is an irreplaceable step in both small‐ and large‐scale manufacturing of cell‐based foods, pMuSCs at P12 to P14 were selected for subsequent experiments. We first investigated the effect of directly co‐culturing pMuSCs with pMSCs and/or porcine smooth muscle cells (pSMCs) on myotube production. Co‐cultures of two or three cell types were performed at varying ratios while maintaining a constant total number of cells. The results showed that co‐culturing pMuSCs with pMSCs and/or pSMCs significantly enhanced the myogenic efficiency of pMuSCs. Specifically, a 7:3 ratio of pMuSCs to pMSCs markedly promoted myotube generation, boosting the percentage of MyHC^+^ nuclei to approximately 17 %, which was 3.5 times higher than that of pMuSCs monoculture (10:0) (Figure 1a,b). When MuSCs and SMCs were co‐cultured, an optimal ratio of 9:1 (pMuSCs: pSMCs) was identified, yielding 14.2 % MyHC^+^ nuclei, 2.7 times higher than that of pMuSC monoculture (10:0) (Figure 1c,d). Subsequently, co‐cultures of pMuSCs, pMSCs, and pSMCs were performed at different cell ratios. As shown in Figure 1e,f, a 7:2:1 ratio of pMuSCs: pMSCs: pSMCs resulted in significantly enhanced myotube generation compared to other ratios and pMuSCs monoculture.

The above data suggest that co‐culturing pMuSCs with pMSCs and/or pSMCs improves myotube formation. To further confirm this finding and determine the optimal co‐culture system, we analyzed the expression of proteins and genes specific to myotubes. As shown in Figure 1g,h, the highest MyHC protein level was found in the co‐culture system of pMuSCs and pMSCs in a 7:3 ratio (pMuSCs: pMSCs: pSMCs = 7:3:0), which was twice as much as in the pMuSC monoculture system (pMuSCs: pMSCs: pSMCs = 10:0:0). Similarly, this co‐culture system showed the highest expression levels of *MYH1*, *MYH2*, *MYH3*, *MYH7*, and *MYOG* genes, confirming superior myogenic differentiation and maturity (Figure 1i). Collectively, these results demonstrate that the co‐culture of pMuSCs with pMSCs (7:3 ratio) is highly effective for promoting myotube formation.

Previous studies have shown that MSCs can secrete a variety of regulatory factors associated with muscle development, such as insulin‐like growth factor‐binding protein (IGFBP)‐7, IL‐6, and matrix metalloprotease 1 (MMP‐1) [22]. To further explore this, we collected the culture supernatant from pMSCs monocultures at different time points and used it as conditioned medium (CM) to culture pMuSCs. As expected, the CM from pMSCs significantly enhanced the myogenic efficiency of pMuSCs, with the CM collected at 36 h demonstrating the most pronounced effect (Figure S2a,b). However, the pro‐myogenic effect diminished when the CM was collected after 48 h, probably due to the accumulation of metabolic wastes that are detrimental to myogenesis. Overall, our results highlight the essential role of co‐culture systems of pMuSCs and pMSCs, either directly or indirectly, in enhancing the myogenic potential of pMuSCs.

### 2.2 Transcriptomic Profiling of pMuSCs in Monoculture and Co‐Culture Systems

**FIGURE 2:**
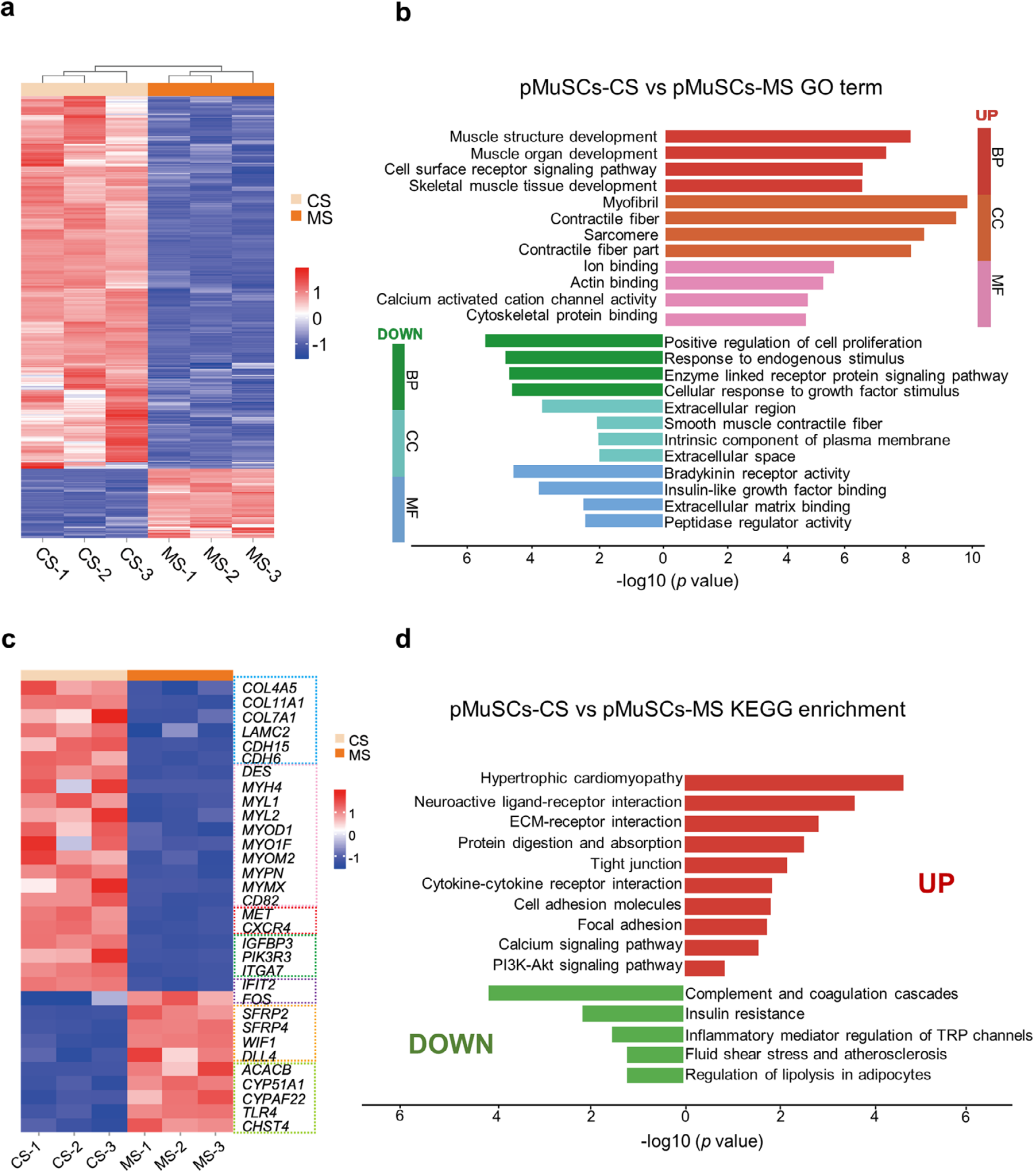
Clustering analysis of differential gene expression patterns between pMuSCs‐CS and pMuSCs‐MS. (a) Heatmap of total genes in pMuSCs‐CS and pMuSCs‐MS. (b) GO enrichment analysis of differential genes between pMuSCs‐CS and pMuSCs‐MS, highlighting up‐regulated biological processes (BP) in red, up‐regulated cellular components (CC) in orange, up‐regulated molecular functions (MF) in pink, down‐regulated BP in green, down‐regulated CC in cyan, and down‐regulated MF in blue. (c) Heatmap showing the expression patterns of genes corresponding to ECM remodeling, myogenic development, cell migration, and cell metabolism. (d) KEGG enrichment analysis of differential genes between pMuSCs‐CS and pMuSCs‐MS. For (b,d), *p*‐values were obtained using a two‐tailed hypergeometric test without adjustment for multiple comparisons.

To elucidate how the co‐culture system regulates myogenic differentiation of pMuSCs, we performed RNA‐seq analysis of pMuSCs in monoculture and co‐culture systems. Correlation analysis showed high reproducibility among the biological replicates, with significant differences between pMuSCs in the monoculture system (MS) and in the co‐culture system with pMSCs (CS). A total of 526 differentially expressed genes (DEGs) were identified in both cell populations, with 443 genes up‐regulated and 83 genes down‐regulated in the pMuSCs‐MS compared to pMuSCs‐CS (Figure 2a; Figure S3a,b). Gene Ontology (GO) enrichment analysis indicated that the pMuSCs‐CS exhibited up‐regulation of functions related to muscle development, myofiber formation, and receptor signaling pathway, suggesting that the pMSCs secreted factors that promote muscle generation. Concurrently, biological functions associated with cell proliferation and responses to growth factor stimulus were down‐regulated, indicating a transition of cells from a proliferative to a differentiated state (Figure 2b). Kyoto Encyclopedia of Genes and Genomes (KEGG) analysis revealed that the up‐regulated genes in pMuSCs‐CS were predominantly associated with the extracellular matrix (ECM), cell adhesion, and Ca^2+^ signaling pathway, which have been shown to play an important regulatory role in myogenic differentiation [23, 24]. Instead, the down‐regulated pathways were primarily associated with coagulation cascades and insulin resistance, which have been shown to contribute to muscle atrophy (Figure 2d).

Furthermore, we analyzed top up‐regulated and down‐regulated genes from the RNA‐seq data (Figure 2c). Significantly up‐regulated genes involved in ECM remodeling (such as *COL4A5*, *COL11A1*, and *LAMC2*), cell surface receptors related to developmental regulation (such as *ITGA7*, *IGFBP3*), and cell migration (such as *MET* and *CXCR4*). Meanwhile, inhibitors of the Wnt signaling pathway, such as *SFRP2*, *SFRP4*, and *WIF1*, were significantly down‐regulated. Further qPCR analysis of the aforementioned representative genes (involving ECM remodeling, cell migration, cell surface receptors, and the Wnt signaling) validated the reliability of the RNA‐seq data (Figure S3c,d). Consequently, pMuSCs‐CS exhibited robust up‐regulation of multiple myogenic transcripts, including the transcription factor *MYOD1*, myosin family genes *MYH4*, *MYL1*, and *MYL2*, as well as the titin family genes *MYOM2* and *MYPN* (Figure S3e). These findings demonstrate that co‐culture with pMSCs promotes myogenesis through coordinated ECM remodeling, myogenic development, and cell migration.

### 2.3 Simultaneous Production of Myotubes and Adipocytes in the Co‐Culture System of pMuSCs and pMSCs

Dual induction of myogenic differentiation and adipogenic differentiation is essential for producing nutritious, flavorful cell‐based foods. Previous studies have shown differentiation of MSCs into adipocytes using inducer cocktails typically consisting of insulin (INS), rosiglitazone (ROS), oleic acid (OA), etc [19, 25, 26, 27]. Since we developed a co‐culture system consisting of pMuSCs and pMSCs in a 7:3 cell ratio, which significantly promoted myotube formation, we hypothesized that the addition of adipogenic inducers to this co‐culture system would yield both myotubes and adipocytes. However, it remained unclear whether the inclusion of adipogenic inducers would inhibit myogenic differentiation of pMuSCs.

**FIGURE 3:**
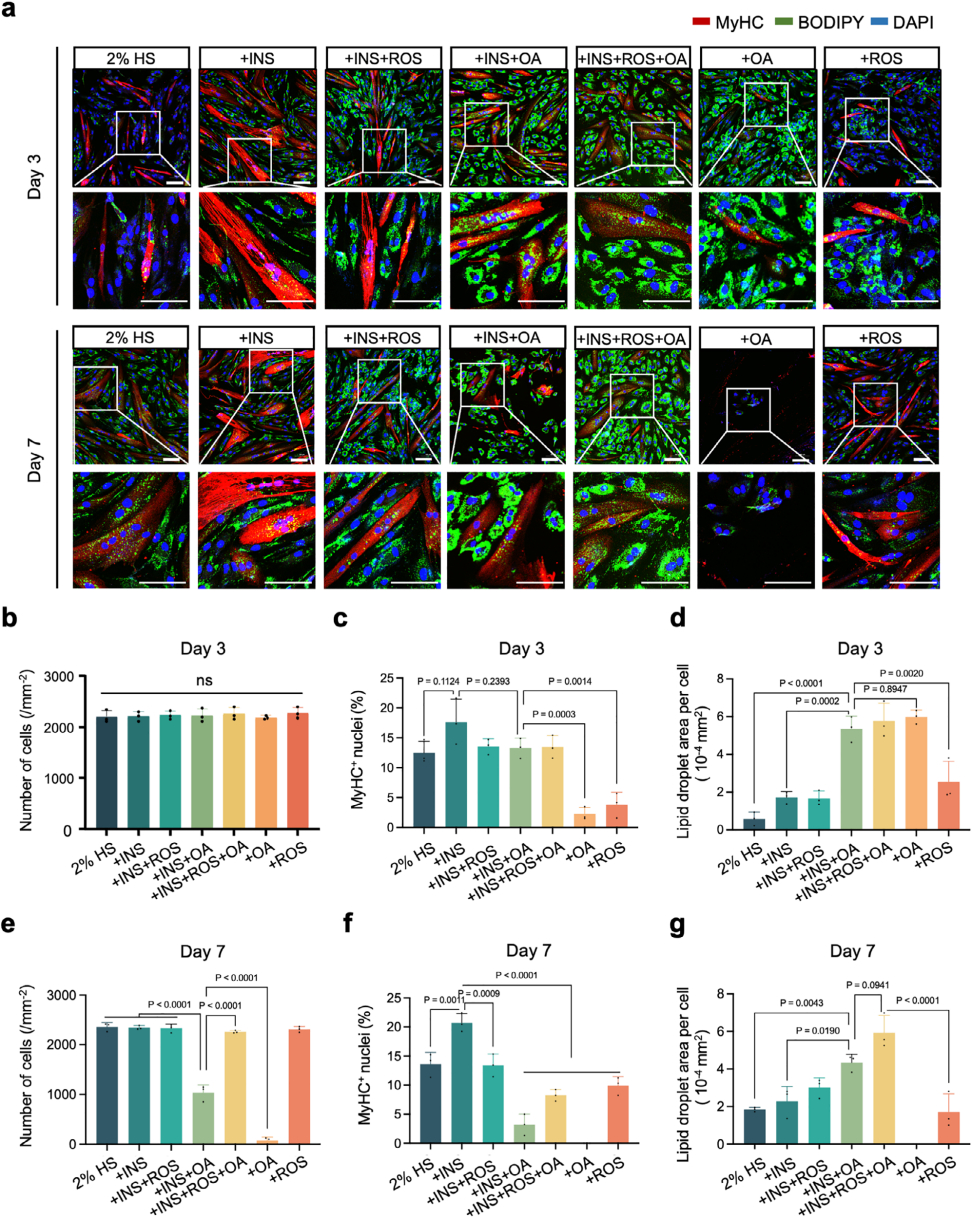
Dual induction of myogenic and adipogenic differentiation in the co‐culture system of pMuSCs and pMSCs. (a) Representative fluorescence images showing MyHC (red) and Bodipy (green) staining in the co‐culture system after 3 and 7 days of induction under different conditions. Nuclei were counterstained with DAPI. Scale bar: 100 µm. (b) Quantification of cell numbers at day 3. (c) Quantification of the percentage of MyHC^+^ nuclei at day 3. (d) Quantification of lipid droplet area at day 3. (e) Quantification of cell numbers at day 7. (f) Quantification of the percentage of MyHC^+^ nuclei at day 7. (g) Quantification of lipid droplet area at day 7. For (a), similar results were obtained in three independent experiments. For (b‐g), error bars indicate means ± SD (n = 3 independent experiments). Significance was determined by one‐way ANOVA with Tukey's post hoc analysis. *p*‐values are annotated in the figures. ns indicates no significant difference (*p* > 0.05).

We first examined the effects of adding INS, ROS, and OA individually or in combination to the basal medium containing 2 % HS on the co‐differentiation of myogenesis and adipogenesis. After 3 days of culture, the cell numbers in each group were comparable, but the formation of myofibers and adipocytes exhibited significant differences in the presence of different inducers (Figure 3a,b). The addition of INS to the co‐culture system markedly enhanced myotube formation, increasing the percentage of MyHC^+^ cells by 40 % compared to the 2 % HS group, while having a slight effect on adipogenesis (Figure 3a,c,d). In contrast, adipogenesis was significantly elevated in all OA‐containing groups, with lipid droplet areas more than 1.8‐fold higher than in the other groups (Figure 3a,d). However, OA alone significantly suppressed myogenic differentiation of pMuSCs (Figure 3a,c). Fortunately, the combination of INS with OA simultaneously yielded robust myotubes and abundant adipocytes within 3 days, achieving well‐balanced co‐differentiation. When the culture was extended to 7 days, no further increase in myotube or lipid droplet formation was observed. Instead, OA‐containing groups showed varying degrees of cell apoptosis and detachment of myotubes or adipocytes (Figure 3a,e). Previous studies have reported that as adipogenesis proceeds, some cells may undergo lipotoxicity due to over‐accumulation of lipids, leading to endoplasmic reticulum stress and mitochondrial dysfunction, ultimately activating apoptotic pathways [28, 29]. The apoptotic factors (e.g., cytochrome c [30], caspase activators [31]) and other pro‐apoptotic signals released from these dying cells can adversely affect other cells in the same culture system, including differentiated myotubes, compromising their integrity and survival. Therefore, extending the culture period compromised co‐differentiation outcomes due to OA‐induced lipotoxicity. A 3‐day induction with combined INS and OA represented a balanced and efficient strategy to achieve simultaneous myogenesis and adipogenesis.

Furthermore, we detected the expression levels of genes and proteins related to myogenesis and adipogenesis after 3 days of culture with different inducers. As expected, the expression of MyHC was significantly increased in cells treated with INS and INS+OA, indicating the generation and maturation of myotubes (Figure S4a,b). Also, the expression of fatty acid binding protein 4 (FABP4) was significantly increased in cells treated with OA and ROS, indicating the generation and maturation of adipocytes (Figure S4a,c). qPCR data involving myogenic genes (*MYH3*, *MYH7*, and *MYOG*) and adipogenic genes (*PPARγ*, *FABP4*, and *PLIN1*) demonstrated similar trends (Figure S4d–i). Collectively, the above data showed that the combination of INS and OA induced simultaneous myogenic differentiation and adipogenic differentiation in the pMuSCs and pMSCs co‐culture system in 3 days.

### 2.4 Development of an Efficient Serum‐Free Co‐Differentiation Medium

**FIGURE 4:**
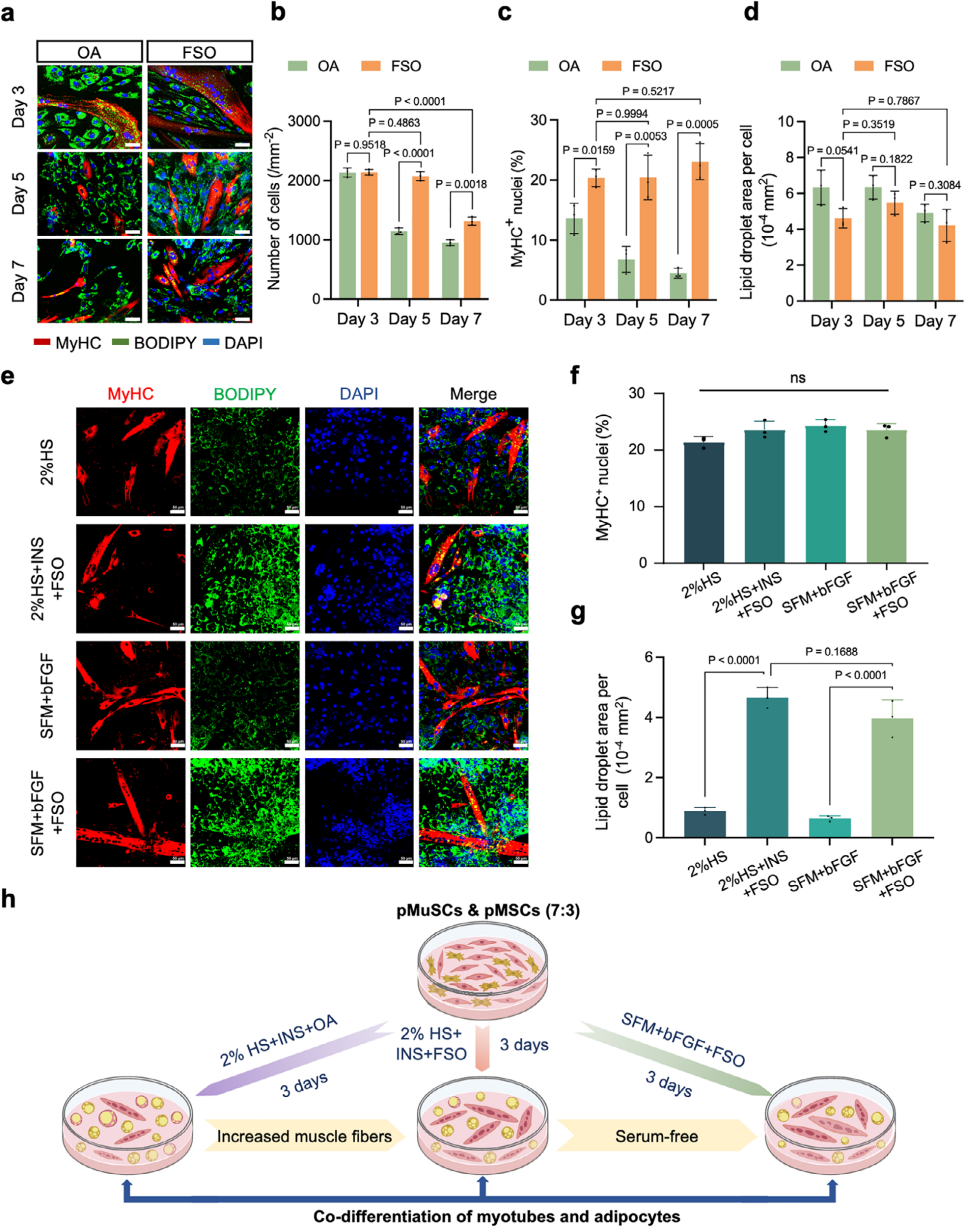
Development of an efficient serum‐free co‐differentiation medium. (a) Representative MyHC (red) and Bodipy (green) fluorescence staining images of the co‐culture system after 3–7 days of differentiation induced by INS+OA or INS+FSO. DAPI was used for nuclear staining (blue). Scale bar: 50 µm. (b) Quantification of cell numbers at different time points in the co‐culture system (n = 3 independent experiments). (c) Quantification of the percentage of MyHC^+^ nuclei at different time points in the co‐culture system (n = 3 independent experiments). (d) Quantification of the lipid droplet area at different time points in the co‐culture system (n = 3 independent experiments). (e) Representative MyHC (red) and Bodipy (green) fluorescence staining images of the co‐culture system after 3 days of differentiation induction using serum‐containing or serum‐free medium. DAPI was used for nuclear staining (blue). Scale bar: 50 µm. (f) Quantification of the percentage of MyHC^+^ nuclei in the co‐culture system after 3 days of differentiation induction using serum‐containing or serum‐free medium (n = 3 independent experiments). (g) Quantification of lipid droplet area in the co‐culture system after 3 days of differentiation induction using serum‐containing or serum‐free medium (n = 3 independent experiments). (h) Schematic diagram of the process flow for developing the serum‐free co‐differentiation medium. For (b–d, f,g), error bars indicate means ± SD. Significance was determined by Student's t‐tests or one‐way ANOVA with Tukey's post hoc analysis. *p*‐values are annotated in the figures. ns indicates no significant difference (*p* > 0.05). For (a,e), similar results were obtained in three independent experiments.

Given the critical role of protein as an indispensable nutrient, we sought to optimize the co‐differentiation induction system to further increase myotube yield. Since OA strongly induced adipogenesis but impeded myogenesis, we hypothesized that replacing OA with a milder adipogenic inducer would improve myotube production. Edible oils contain a diverse array of fatty acids that have been reported to induce adipogenesis and significant benefits such as cost‐effectiveness, accessibility, and food safety [32]. Here, we selected 5 common edible oils, including peanut oil (PO), sunflower seed oil (SSO), perilla seed oil (PSO), camellia seed oil (CSO), and flaxseed oil (FSO), and investigated their effects on myogenesis and adipogenesis in the co‐culture system. Among them, FSO at 0.5 µL/mL showed relatively high adipogenic efficiency without compromising myogenesis (Figure S5a–d). We next evaluated the impact of replacing OA with FSO in the co‐differentiation system. The results indicate that no noticeable cell detachment occurred during the first 5 days with FSO induction, but partial cell apoptosis or detachment was still observed by day 7 (Figure 4a,b). On day 3, FSO yielded a significantly higher proportion of MyHC^+^ nuclei than OA, despite a slight decrease in lipid droplet area, but no statistical difference (Figure 4c,d). Prolonging FSO treatment to day 5 did not significantly enhance myotube or lipid formation (Figure 4a,c,d). Because a shorter, simpler process is preferable for industrial production, we conclude that a 3‐day induction with INS and FSO offers the optimal biological and operational balance, and therefore adopted this approach for subsequent research.

The development of serum‐free media is crucial for reducing batch variability and addressing animal ethics concerns, also lowering culture costs. We previously developed a serum‐free medium (SFM) that efficiently supported the survival and induced the differentiation of pMuSCs into myotubes, which was DMEM supplemented with INS, transferrin, selenium, BSA, and MEM amino acids [33]. Unfortunately, this SFM failed to maintain pMSC adhesion and viability, causing significant cell detachment after 24 h of culture (Figure S6a). To optimize SFM for pMSC survival and growth, we added four growth factors (bFGF, EGF, IGF‐1, and TGF‐β) individually and in combination. Cytokine addition, particularly bFGF, markedly improved pMSC viability, maintaining it above 80 % of the level achieved with 2 % HS (Figure S6b). Importantly, adding bFGF to SFM enhanced pMuSC myogenesis efficiency compared to the conventional 2 % HS medium (Figure S6c,d). Since SFM already contained INS, we further supplemented SFM with bFGF and FSO to develop a serum‐free co‐differentiation medium. As shown in Figure 4e–g, this newly developed serum‐free co‐differentiation medium (SFM + bFGF + FSO) effectively induced simultaneous myotube and adipocyte generation, performing comparably to the serum‐containing medium (2 % HS + INS + FSO).

Taken together, starting from a serum‐containing co‐differentiation medium with inducers INS and OA, we developed a serum‐free co‐differentiation medium by replacing OA with FSO and adding serum substitutes along with bFGF (Figure 4h). This serum‐free co‐differentiation medium effectively supported both myogenic and adipogenic differentiation in 3 days, yielding muscle proteins and lipids.

### 2.5 Physical Properties and Cytocompatibility of Starch‐Based Scaffolds

**FIGURE 5:**
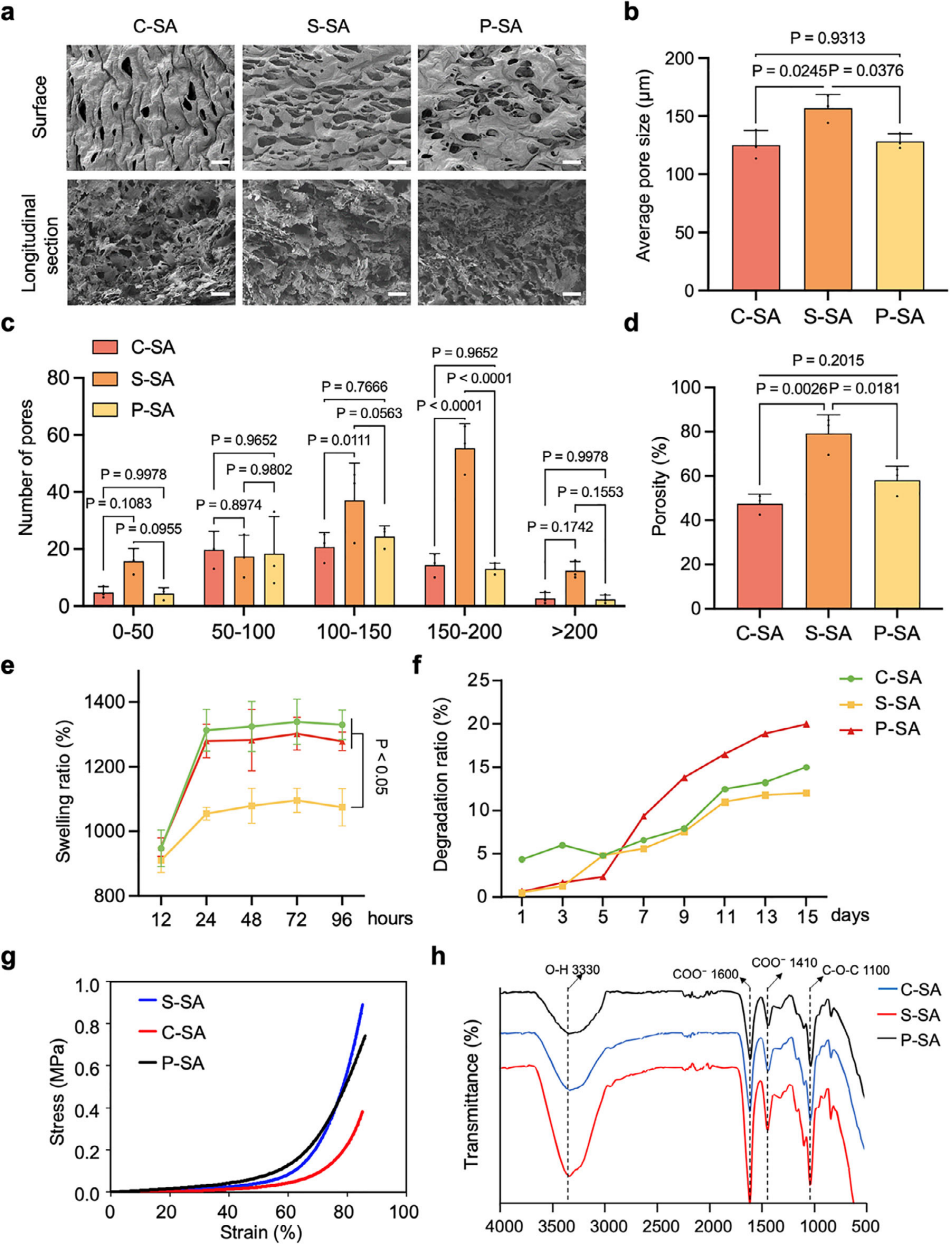
Examination of the structure and physical properties of starch‐based scaffolds. (a) Representative SEM images of C‐SA, S‐SA, and P‐SA scaffolds. Scale bar: 200 µm. (b) Average pore size of C‐SA, S‐SA, and P‐SA scaffolds (n = 3 independent experiments). (c) The pore size distribution of C‐SA, S‐SA, and P‐SA scaffolds (n = 3 independent experiments). (d) The porosity of C‐SA, S‐SA, and P‐SA scaffolds (n = 3 independent experiments). (e) The swelling ratio of C‐SA, S‐SA, and P‐SA scaffolds at different time points (n = 3 independent experiments). (f) The degradation ratio of C‐SA, S‐SA, and P‐SA scaffolds during 15 days of culture. (g) The stress–strain curve of C‐SA, S‐SA, and P‐SA scaffolds (n = 3 independent experiments). (h) FTIR of C‐SA, S‐SA, and P‐SA scaffolds. For (b–e), error bars indicate means ± SD. Significance was determined by one‐way ANOVA with Tukey's post hoc analysis. *p*‐values are annotated in the figures. For (a, f–h), similar results were obtained in three independent experiments.

We next explored the production of cell‐based hybrid noodles by co‐culturing pMuSCs and pMSCs on starch‐based scaffolds. We selected commonly consumed starches, i.e., sweet potato starch (S), corn starch (C), and potato starch (P), and fabricated three kinds of starch‐based scaffolds by combining them with sodium alginate (SA) via calcium ion cross‐linking. Scanning electron microscopy (SEM) images showed that the S‐SA scaffolds had more pores with an organized and interconnected pore network compared to the C‐SA and P‐SA scaffolds (Figure 5a). Also, the S‐SA scaffold provided the largest average pore size, predominantly in the 100–150 µm and 150–200 µm ranges (Figure 5b,c). This aligns with prior studies suggesting that composite scaffolds with pore sizes of 85–190 µm were ideal for cell attachment and growth [34]. Also, the porosity of the S‐SA scaffold was more than 70 %, remarkably greater than the other two scaffolds (Figure 5d).

Furthermore, the mechanical properties and stability of different starch‐based scaffolds were tested after immersion in the medium. The S‐SA scaffold exhibited significantly lower swelling ratio than the other two scaffolds (Figure 5e), as well as lower degradation ratio during long‐term incubation at 37°C in culture medium (Figure 5f). Moreover, the mechanical strength of three kinds of scaffolds was determined by a compression test. As shown in Figure 5g, the S‐SA scaffold showed the greatest compressive strength of about 0.89 MPa, which was slightly higher than the P‐SA scaffold and significantly higher than the C‐SA scaffold. Additionally, the stronger interactions between S and SA in the S‐SA scaffold were determined by Fourier Transform Infrared Spectroscopy (FTIR) (Figure 5h). All these results together demonstrated the excellent mechanical strength and stability of the S‐SA scaffold.

**FIGURE 6:**
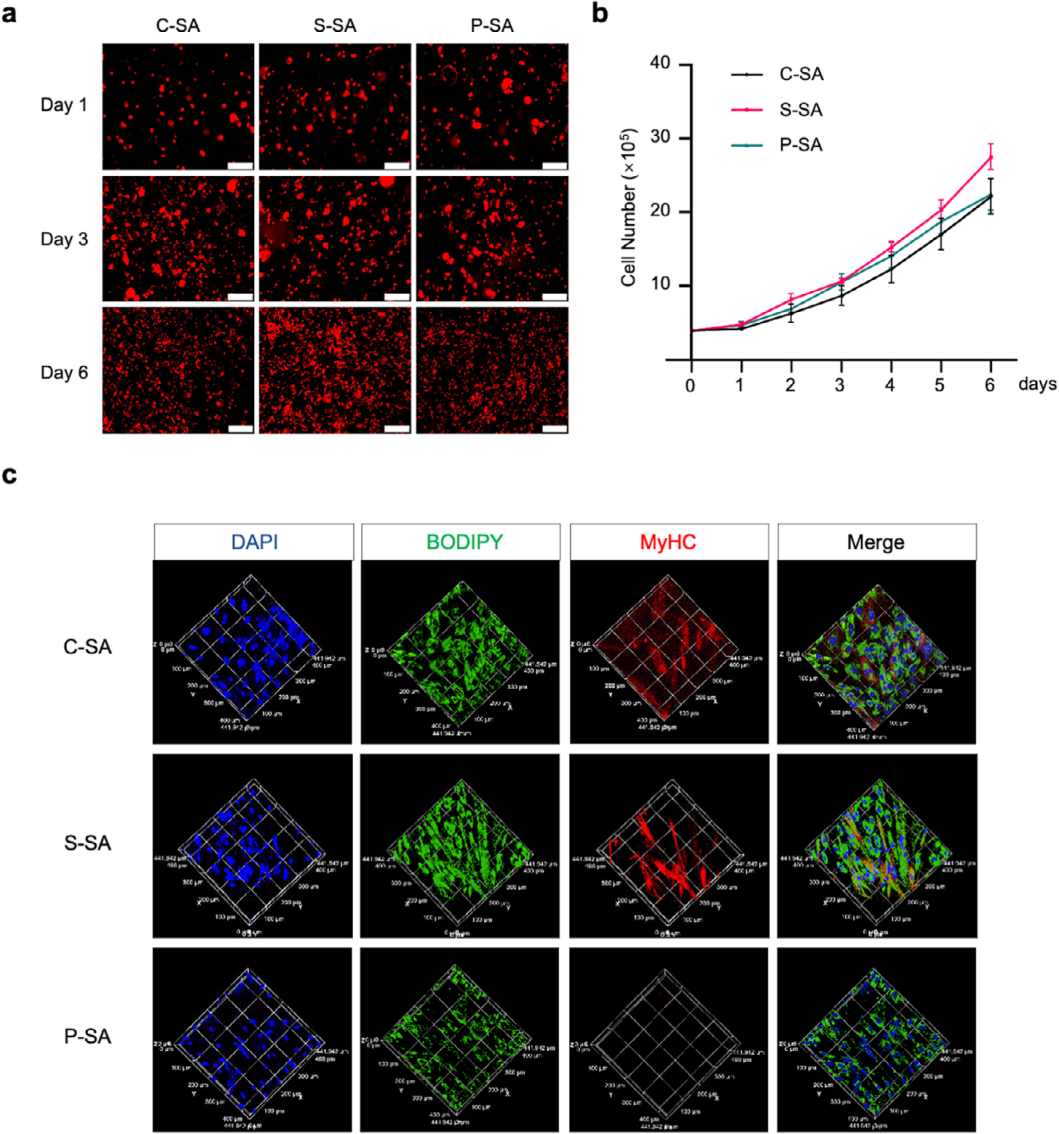
Examination of the cytocompatibility of starch‐based scaffolds. (a) Representative fluorescence images of Dil‐labeled cells after 1, 3, and 6 days of culture on C‐SA, S‐SA, and P‐SA scaffolds. Scale bar: 200 µm. (b) The number of cells on C‐SA, S‐SA, and P‐SA scaffolds (n = 3 independent experiments). (c) Representative MyHC (red) and Bodipy (green) fluorescence staining images after 3 days of co‐differentiation of pMuSCs and pMSCs on C‐SA, S‐SA, and P‐SA scaffolds. DAPI was used for nuclear staining. For (b), error bars indicate means ± SD. For (a,c), similar results were obtained in three independent experiments.

We next assessed the scaffold cytocompatibility by examining the adhesion, proliferation, and differentiation of co‐cultured pMuSCs and pMSCs. All scaffolds were non‐significantly toxic and supported rapid cell adhesion, as confirmed by >80 % cell survival in the leaching assay and >90 % adhesion at 6 h of cell seeding (Figure S7a,b). Then, pMuSCs and pMSCs were mixed, labeled with the cell membrane dye Dil, and seeded onto three starch‐based scaffolds, respectively. Fluorescence imaging revealed uniform cell distribution and proliferation, with S‐SA scaffolds achieving the highest cell density (2.79 × 10^6^ cells/cm^3^ by day 6; Figure 6a,b). Consistently, F‐actin filaments were visualized by fluorescent phalloidin staining after 6 days of culture, which showed that the cells were well‐spread in the scaffolds and almost entirely filled the scaffolds (Figure S7c). Furthermore, the differentiation‐supporting effects of the three scaffolds were evaluated. Surprisingly, there were significant differences in the generation of myotubes and adipocytes on the three scaffolds (Figure 6c). MyHC^+^ myotubes were barely visible, and lipid droplets were minimal on the P‐SA scaffold. Although both S‐SA and C‐SA scaffolds showed good performance in supporting adipogenesis, the S‐SA scaffold presented better effects in supporting myotube generation.

Taken together, the above data suggest that starch can be engineered into scaffolds with desirable pore structure, durability, and cytocompatibility, thus creating a conducive environment for pMuSCs and pMSCs to adhere and proliferate. Among these three scaffolds, the S‐SA scaffold stands out for its superior porosity, pore size, and mechanical strength, yielding a promising result in promoting both myogenic and adipogenic differentiation in the co‐culture system.

### 2.6 Nutrition and Flavor Profiles of Cell‐Based Hybrid Noodles

**FIGURE 7:**
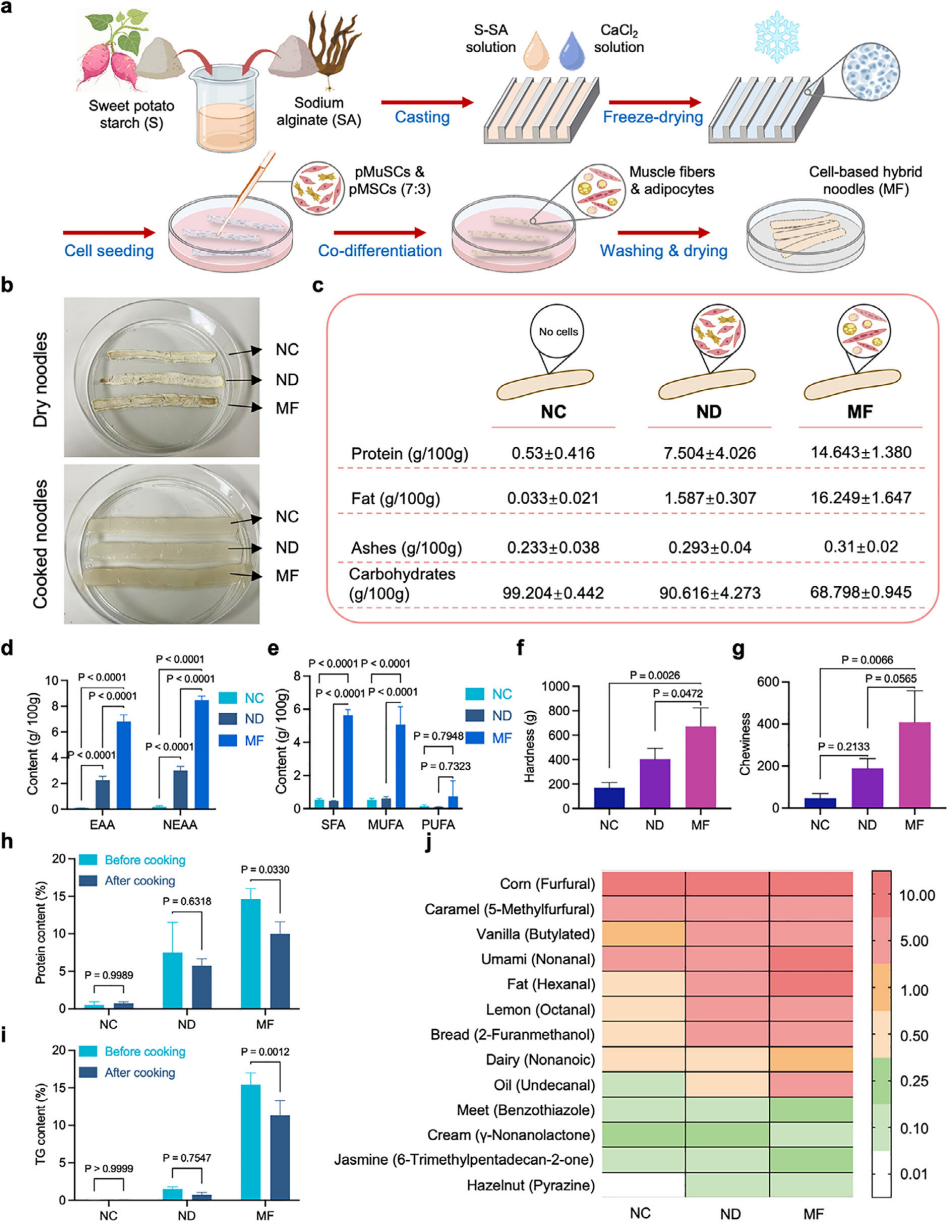
Nutritional and flavor assessment of cell‐based hybrid noodles. (a) Schematic diagram of the manufacture of cell‐based hybrid noodles. (b) The appearance of dry and cooked cell‐based hybrid noodles. NC indicates pure S‐SA noodles without cells, ND indicates hybrid noodles containing pMuSCs and pMSCs, and MF indicates hybrid noodles containing myotubes and adipocytes. (c) Nutritional analysis of NC, ND, and MF (n = 3 independent experiments). (d) EAA and NEAA content of NC, ND, and MF (n = 3 independent experiments). (e) PUFA, MUFA, and SFA content of NC, ND, and MF (n = 3 independent experiments). (f) Hardness of NC, ND, and MF (n = 3 independent experiments). (g) Chewiness of NC, ND, and MF (n = 3 independent experiments). (h) Protein content of NC, ND, and MF before and after cooking (n = 3 independent experiments). (i) TG content of NC, ND, and MF before and after cooking (n = 3 independent experiments). (j) A heatmap illustrating the flavor intensity of NC, ND, and MF. The color intensity indicates the peak area of the compound detected by GC‐MS. For (d–g), error bars indicate means ± SD. Significance was determined by one‐way ANOVA with Tukey's post hoc analysis. *p*‐values are annotated in the figures. For (b), similar results were obtained in three independent experiments.

By co‐culturing pMuSCs and pMSCs on starch‐based scaffolds and inducing simultaneous myogenic and adipogenic differentiation, followed by washing and drying, we successfully created cell‐based hybrid noodles (Figure 7a). To evaluate the impacts of animal stem/progenitor cells (undifferentiated) and muscle/fat cells (post‐differentiation) on the nutritional profile, texture, and flavor of hybrid noodles, three sets of samples were bio‐fabricated and examined: pure S‐SA noodles without cells (NC), hybrid noodles containing co‐cultured pMuSCs and pMSCs (ND), and hybrid noodles containing differentiated porcine myotubes and adipocytes (MF). In dried form, it can be observed that compared to the NC sample, ND and NF samples exhibit irregular textures and uneven colors owing to the presence of cells. In particular, the NF sample displayed a distinct dark brown color and a more coarse texture (Figure 7b). Nutritional analysis revealed that, similar to traditional noodles, the NC samples served as a carbohydrate‐dominated control with minimal protein and fat content. The incorporation of animal cells markedly elevated the protein and fat content in the NF and MF samples. Most notably, the MF sample exhibited a significantly higher protein content (14.643 ± 1.380 g/100 g), indicating its potential to meet the body's protein requirement. Moreover, the fat content of MF noodles was 16.249 ± 1.647 g/100 g, which was significantly higher than that of NC and ND, implying a richer texture and flavor (Figure 7c).

On analyzing the amino acid profiles of three sets of samples, we found that MF had about twice the amount of essential amino acid (EAA) content compared to ND (Figure 7d). Specifically, the levels of branched‐chain amino acid (BCAAs), including Valine (Val), leucine (Leu), and isoleucine (Ile), were 2–3 times higher in MF than in ND (Figure S8a). These data suggest that the MF samples underwent efficient myogenic differentiation to generate myotubes, given that these amino acids are abundant in muscle proteins [35]. In terms of the fatty acid profile, 24 fatty acids were detected in the samples from both the ND and MF groups (Figure S8b). Meanwhile, no trans fatty acids, such as lanoleic acid or trans linoleic acid, which may increase the risk of cardiovascular disease, were detected. Notably, the contents of PUFA, MUFA, and SFA in MF were approximately 12.2, 8.2, and 6.3 times higher than those in ND, respectively, mainly attributed to the efficient adipogenic differentiation (Figure 7e).

Given that boiling is the standard cooking method for noodles, all three sets of samples were boiled in 100°C water for 5 min. After cooking, all noodles absorbed water and swelled, and the MF sample showed a darker color than the other two groups (Figure 7b). Moreover, the water absorption of ND and MF samples was significantly lower than that of NC samples, implying cells might have compacted the noodle structure (Figure S8c). To identify if cells in hybrid noodles detached during cooking, we measured the protein and fat content of different noodle samples before and after boiling. The results showed that the cooking losses of protein and triglycerides (TG) in the MF samples were less than 25 % (Figure 7h,i), suggesting that cell‐based hybrid noodles can better retain their cell components and nutrients during the cooking process. Additionally, each textural property, such as hardness, springiness, and chewiness, was significantly elevated in ND and MF compared to NC (Figure 7f,g; Figure S8d–g), indicating that the cell loading enhances noodle mechanical strength and structural stability during cooking. Notably, the MF samples demonstrated approximately 65.7 % greater hardness, 116.6 % higher chewiness, and 78.2 % higher gumminess than the ND samples. These data, consistent with water absorption results, suggest that the myotube bundles and ECM proteins from cell differentiation form a dense network, giving hybrid noodles a chewier texture.

To further explore the flavor characteristics of cell‐based hybrid noodles, we conducted a qualitative and quantitative analysis of three sets of cooked noodle samples (Figure 7j). Typical cereal and plant flavor compounds were detected across all three sample sets, including furfural (reminiscent of sweet corn), 5‐methylfurfural (characteristic of caramel), and butylated (related to vanilla), which was likely linked to the background flavor of the S‐SA scaffold. Significant increases in meat‐related compounds were shown in the ND and MF samples, suggesting that incorporating animal cells induces a significant shift in the flavor profile of the hybrid noodles. Of particular interest was the MF sample, which showed a marked abundance of compounds linked to umami (nonanal), fat (hexanal), oil (undecanal), and meaty aromas (benzothiazole), suggesting that this muscle and fat‐containing hybrid noodle exhibits a more meaty flavor than its pure starch counterparts. Taken together, these results demonstrate that cell‐based hybrid noodles not only offer enhanced nutritional value and improved texture but also provide a more complex and meaty flavor profile compared to traditional starch‐based noodles. It also highlights the potential of cell‐based hybrid foods to fulfill the nutritional and organoleptic requirements of consumers.

## 3 Discussion

Cell‐based food technologies are transforming animal‐derived food production, offering environmental, standardization, and animal welfare benefits. While current efforts predominantly focus on cell‐based meat, replicating real meat's sensory and nutritional qualities, especially in complex cuts like steaks, remains challenging. In contrast, cell‐based hybrid foods present several advantages. By integrating animal cell‐derived proteins and fats with carbohydrate‐rich staple foods, they can achieve a more balanced nutritional profile at a potentially lower cost. Furthermore, unlike cell‐based meat, which faces high consumer expectations, hybrid foods provide a novel product experience that mitigates direct comparisons with traditional meat. This novel category may attract a broader audience, including consumers who remain hesitant about cell‐based meat. Therefore, cell‐based hybrid foods represent a pioneering pathway to introduce cellular agriculture technologies to consumers.

From a food perspective, efficient biosynthesis of muscle protein via cell culture technology holds great significance, given the nutritional value of animal protein and the sustainability challenges of conventional livestock farming. To meet the cell biomass requirements for scalable production, it is necessary to use cells at late passages rather than early passages. However, prolonged in vitro expansion often compromises the myogenic potential of MuSCs. Therefore, in this work, we purposely employed pMuSCs at P12–P14, whose myogenic potential drops to <7 % under 2 % HS conditions (Figure S1b). Co‐culture systems offer a promising solution to promote cell differentiation, as animal muscle itself is a complex tissue composed of ECM and diverse cells. Previous studies showed that co‐culturing bovine MuSCs with other cell types enhances myogenesis and ECM deposition [36]. Inspired by this, we explored the effects of co‐culturing pMuSCs with pSMCs and/or pMSCs on myotube formation. We selected these two cell types because they both produce ECM, and pMSCs also have strong adipogenic potential. Our results showed that co‐culturing pMuSCs with either pSMCs or pMSCs significantly improved myotube formation, highlighting the importance of cell interactions. Surprisingly, the highest myotube yield was achieved not in the tri‐culture system but in the co‐culture system consisting of pMuSCs and pMSCs in a 7:3 ratio (Figure 1g–i). RNA‐seq analysis revealed that co‐culturing with pMSCs enhances myogenesis through the regulation of ECM production and multiple signaling pathways (Figure 2d). Additionally, we found that myogenic efficiency dropped when the pMuSCs proportion fell below 50 % (Figure 1a–d), likely due to altered cell interactions. These findings highlight the importance of cell types and ratios in the process of in vitro myogenesis and maturation.

Developing efficient muscle‐fat co‐differentiation systems remains a key challenge in cell‐based food technology, due to the distinct inducers and timelines for myogenic and adipogenic differentiation. Conventional co‐culture strategies, as reported in previous studies, typically rely on serum‐containing media and separate myogenic and adipogenic differentiation formulations, resulting in a culture cycle of 7–10 days [20, 37]. Moreover, some protocols depend on chemical inducers such as IBMX or ROS, raising safety and regulatory hurdles for food‐grade applications [38]. Our research group has invested efforts in developing co‐differentiation media that accommodate both myogenic and adipogenic differentiation requirements. Previously, we developed a co‐differentiation medium containing 2 % HS, adipogenic inducer cocktails A/B, and OA, enabling simultaneous directional differentiation of pMuSCs and pAMSCs in 7 days [19]. In this work, we found that a simple combination of insulin and lipids with 2 % HS yielded both myotubes and adipocytes in a 3‐day culture, greatly simplifying the previous formulation. OA has been reported to strongly drive adipogenesis via DGAT2‐mediated triglyceride synthesis and PPARγ activation [39]. However, this strong adipogenic effect resulted in impaired myogenesis of pMuSCs in the co‐culture system (Figure 3). Therefore, we screened a variety of edible oils, trying to find one with a pro‐adipogenic effect similar to that of OA but without compromising myogenesis. Fortunately, we identified FSO, a commonly used vegetable oil, as a promising candidate. The α‐linolenic acid in FSO is an important plant‐derived ω‐3 fatty acid, which can activate PPARγ and thus induce adipogenesis [40]. Moreover, FSO contains lignans, which have been reported to synergistically promote myotube fusion by activating the AMPK pathway [41]. Additionally, compared to OA, vegetable oils are more readily available and cost‐effective. Thus, FSO offers a practical and economical inducer for promoting both adipogenesis and myogenesis in the co‐culture system. Additionally, the efficacy of this optimized co‐differentiation system was further validated using early‐passage cells (P4 and P6), which exhibited robust myogenesis and adipogenesis after 3 days of culture (Figure S9). This validation, together with the data from late‐passage cells, suggests the broad applicability and reliability of our co‐differentiation system across different experimental contexts relevant to cellular agriculture.

Starch serves as both the main ingredient and cell scaffold in our hybrid noodles. Although a variety of scaffolds have been developed for manufacturing cell‐based foods, only a minority are completely “animal‐free”, with most incorporating animal‐derived components like gelatin [18, 42]. Benefiting from the excellent cytocompatibility of starch [43], our starch‐based scaffolds were free of animal‐derived components yet effectively supported cell adhesion and proliferation. Particularly, the S‐SA scaffold showed suitable pore size and porosity, higher mechanical strength, and lower swelling, thereby providing a conducive microenvironment for myogenic and adipogenic differentiation. The ratio of amylose to amylopectin significantly influences the water absorption and swelling behavior of the scaffold [44]. In sweet potato starch, the ratio of amylose to amylopectin is approximately 2:8. This property ensures the mechanical strength of the S‐SA scaffold while facilitating water and nutrient transport, ultimately promoting good cell attachment, proliferation, and differentiation.

Finally, by integrating the developed 3D muscle‐fat co‐differentiation technique with the S‐SA scaffolds, we successfully fabricated cell‐based hybrid noodles incorporating meat ingredients. By comparing the dry mass of cell‐based hybrid noodles with that of the cell‐free control group, we preliminarily assessed the relative contribution of cellular components to the product mass of hybrid noodles. It was found that cellular biomass and associated ECM accounted for approximately one‐third of the total dry mass. By macronutrient analysis, we confirmed a significant increase in protein and lipid content in the final cell‐based hybrid noodles (MF), supporting the practical relevance of our co‐differentiation protocol. Moreover, this hybrid noodle is a nutrient‐balanced product consisting of about 68.8 % carbohydrates, 14.6 % proteins, and 16.3 % fats, which qualifies as a healthy diet. Similar to animal‐derived foods such as beef and whey proteins—known for their high essential amino acid and BCAA content—this hybrid noodle promises to offer high protein utilization and significant nutritional benefits for muscle growth and athletic performance enhancement. More appealingly, this hybrid noodle offers a distinct meaty flavor and chewy texture compared to conventional starch noodles, which would receive a favorable response from consumers. Looking forward, essential characterization regarding micronutrient composition, taste profiling, and digestibility remains to be completed. Furthermore, the influence of cell maturity on processing and final product performance is also a research topic we are actively exploring.

## 4 Conclusions

In conclusion, our study highlights not only the effectiveness of cell co‐culture technology but also demonstrates the potential of combining animal cells and plant ingredients to develop hybrid foods. It advances sustainable and nutritious food systems, offering an appealing option for future food consumption. From an industrial scalability perspective, further efforts will focus on increasing the economic viability of the culture medium and scaling up the 3D differentiation process. The serum‐free and chemical‐inducer‐free medium developed here offers a clear advantage for scalable production. Although additional cost reduction is possible, the current formulation remains feasible for pilot‐scale process development. Moreover, we are actively designing a perfusion‐based bioreactor system compatible with the edible S‐SA scaffold. Key engineering challenges, such as bioreactor configuration, perfusion parameters, and scalable cell‐seeding methods, are currently being addressed to facilitate future transition into large‐scale production.

## 5 Experimental Section

### 5.1 Ethics Statements

All of the animal slaughter experiments were conducted in accordance with the Animal Care and Use Guidelines of Jiangnan University and were approved by the Institutional Animal Care and Use Committee of Jiangnan University (JN. No20211030p0020301[431]).

### 5.2 Isolation and Culture of pMuSCs, pMSCs, and pSMCs

pMuSCs and pSMCs were isolated from 7‐day‐old pigs sourced from a local breeding facility, as previously described [45, 46]. For isolating pMuSCs, the longissimus dorsi muscle tissue was harvested, disinfected with 75 % ethanol, and washed with phosphate‐buffered saline (PBS) containing 1 % antibiotic–antimycotic (AA, Gibco, USA) and 1 % gentamicin (GEN, Sigma–Aldrich, USA). After removing connective tissues, the tissue was minced into 1 mm^3^ pieces and digested with a protease combination consisting of pronase (1 mg/mL, Roche, Switzerland) and dispase II (1 mg/mL, Roche, Switzerland) at 37°C for 90 min. After digestion, the mixture was filtered through 70 and 40 µm cell strainers, and then centrifuged at 1500 rpm for 10 min. The cell pellet was resuspended in the growth medium (GM) comprising Dulbecco Modified Eagle's Medium (DMEM; Gibco, USA) with 10 % fetal bovine serum (FBS; Gibco, USA) and 1 % penicillin‐streptomycin (P/S, Thermo Scientific, USA), and then seeded onto type I collagen‐coated plates. Following a 0.5 h incubation at 37°C, the cells were transferred to Matrigel (Corning Inc., USA)‐coated plates to obtain purified pMuSCs.

For isolating pSMCs, the arterial vessels were harvested and stripped of surface fat and connective tissue. After longitudinal sectioning and scraping off the endothelial layer, the tissue was cut into 1 mm^3^ pieces. These pieces were placed onto 6‐well plates and incubated inverted at 37°C for 4 h. Then, the plate was inverted, and GM was added for further culture. After 7–10 days, once spindle‐shaped pSMCs migrated from the tissue margins and reached 80 % confluence, the tissue pieces were discarded, and cells were passaged.

pMSCs were isolated from the subcutaneous adipose tissue of adult pigs sourced from a local market. After disinfecting with 75 % ethanol and washing with PBS, the tissue was minced and digested with type I collagenase (1 mg/mL, Gibco, USA) at 37°C for 75 min. The digested tissue was filtered through 70 and 40 µm cell strainers and centrifuged to obtain pMSCs. Then, the cells were resuspended in GM and seeded onto culture plates.

The isolated pMuSCs, pMSCs, and pSMCs were all expanded in GM at 37°C in a humidified atmosphere with 5 % CO_2_. Specifically, pMuSCs were cultured in flasks coated with Matrigel, while pMSCs and pSMCs were cultured in uncoated flasks.

### 5.3 Direct Co‐Culture Protocol

In co‐culture systems of two cell types, pMuSCs (P12–P14) were mixed with pMSCs (P8–P12) or pSMCs (P8–P12) at ratios of 0:10, 5:5, 7:3, 9:1, and 10:0. In co‐culture systems of three cell types, pMuSCs, pMSCs, and pSMCs were mixed at ratios of 5:4:1, 6:3:1, 7:2:1, 10:0:0, 0:10:0, and 0:0:10. The cell mixtures were seeded onto 48‐well plates at a total cell number of 5 × 10^4^/ well. Then, cells were cultured in myogenic differentiation medium (MDM) consisting of DMEM supplemented with 2 % horse serum (HS; Gibco, USA) and 1 % P/S for 3 days.

### 5.4 Indirect Co‐Culture Protocol

pAMSCs (P8–P12) were seeded in T‐75 culture flasks at a density of 2 × 10^6^ cells/flask and expanded in GM to reach 80 % cell confluence. Then, the cells were rinsed twice with PBS, and MDM was added to the flasks. After 12, 24, 36, 48, and 60 h, respectively, the culture supernatant was collected and centrifuged to remove cellular debris to obtain conditioned medium (CM). Subsequently, pMuSCs (P12–P14) were cultured in various CM samples for 3 days.

### 5.5 Co‐Differentiation Medium Development

First, the co‐differentiation medium was developed based on the serum‐containing basal medium (MDM). pMuSCs (P12–P14) and pMSCs (P8–P12) were mixed at a ratio of 7:3 and cultured in GM for 12 h. Then, the medium was replaced with MDM with the addition of one or more compounds from 100 nM insulin (INS), 5 µM rosiglitazone (ROS), 0.1 µL/mL (v/v) OA, and cultured for 3 or 7 days. Then, the total cell number and the yield of myotubes and adipocytes were analyzed by immunofluorescence staining, thus determining the suitable co‐differentiation medium and culture time for inducing the simultaneous generation of myotubes and adipocytes. To optimize the co‐differentiation medium, different concentrations of edible oils including peanut oil (PO), sunflower seed oil (SSO), perilla seed oil (PSO), and camellia seed oil (CSO), and flaxseed oil (FSO) were used to substitute for OA, and the optimal edible oil and working concentration were determined based on the yield of myotubes and adipocytes.

To develop the serum‐free co‐differentiation medium, we first supplemented our previously reported serum‐free myogenic differentiation medium (SFM, listed in Table S2) [33] with 10 ng/mL bFGF, 50 ng/mL EGF, 50 ng/mL IGF‐1, 10 ng/mL TGF‐β, or a combination of these four growth factors. The information on recombinant proteins is provided in Table S1. The optimal formulation was then determined based on cell survival and growth. Subsequently, we added the previously selected edible oil to the optimized SFM and evaluated its ability to induce myogenic and adipogenic differentiation of the co‐cultured cells.

### 5.6 Immunofluorescence Staining

The cell samples were fixed with 4 % paraformaldehyde (Sigma, Cat# P1110) at room temperature for 15 min, followed by permeabilization using 0.1 % Triton X‐100 (Sigma, Cat# T8787) for 10 min. After that, they were blocked with 1 % (w/v) bovine serum albumin (BSA; Sigma, Cat# SRE0096) for 30 min. To determine myotubes, the cell samples were incubated with the MyHC antibody (anti‐MYH 3; 1:500; Santa Cruz Biotechnology, CA, USA) at 4°C for 12 h. Following washing twice with PBS, the cell samples were incubated with fluorescently‐labeled secondary antibody, CoraLite488‐conjugated Goat Anti‐Mouse IgG (H+L) (1:1000; Proteintech Group, Wuhan, China) or CoraLite594‐conjugated Goat Anti‐Mouse IgG (H+L) (1:1000; Proteintech Group, Wuhan, China) at 37°C for 2 h. To determine adipocytes, the cell samples were incubated with BODIPY 493/503 (1:500; Invitrogen, USA) at room temperature for 30 min. Subsequently, the samples were washed with PBS and counterstained with DAPI (1:1000; Sigma–Aldrich, USA) at room temperature for 5 min. After that, the samples were washed three times with PBS and observed with a laser scanning confocal microscope (Nikon A1, Japan). At least three fields of view were captured for each sample. The information on antibodies is provided in Table S1.

The Image J software was employed to calculate the percentage of MyHC‐positive nuclei or the lipid droplet area per cell. The percentage of MyHC‐positive nuclei was calculated by dividing the number of nuclei within MyHC‐positive cells by the total number of nuclei. The lipid droplet area per cell was calculated by dividing the total area of lipid droplets by the number of cells in the field of view.

### 5.7 Oil Red O Staining

Oil Red O staining was performed according to the instructions of the Oil Red O Staining Kit (Solarbio, China). Briefly, the cell samples were washed with PBS and incubated with the Oil Red O working solution at room temperature for 30 min. Then, the cells were washed with PBS and counterstained with the hematoxylin staining solution at room temperature for 5 min. The samples were visualized using the microscope (Mshot, Guangzhou, China). At least three fields of view were captured for each sample. The Image J software was employed to calculate the lipid droplet area per cell by dividing the total lipid droplet area by the cell count in the field of view.

### 5.8 Western Blot

The cell samples were collected and lysed on ice for 20 min using RIPA buffer (Beyotime, China). The protein content was quantified with a BCA protein assay kit (Beyotime, China). Proteins were then separated via 10 % SDS‐PAGE and transferred to a PVDF membrane. Following a 1 h blocking with the blocking buffer (Beyotime, Shanghai, China), the membranes were incubated with primary antibodies at 4°C for 12 h. The primary antibodies utilized in this study included rabbit anti‐FABP4 (1:1000, MedChemExpress, USA), mouse anti‐MYH3 (1:1000, Santa Cruz, China), and mouse anti‐GAPDH (1:1000, Proteintech Group, China). Then, the membranes were incubated with HRP‐conjugated secondary antibodies (goat anti‐rabbit IgG or goat anti‐mouse IgG, 1:10 000, Proteintech Group, China) for 2 h at room temperature. Blots were detected using the Tanon 4600SF scanner system (Shanghai, China) and analyzed with ImageJ for densitometry. Protein expression was normalized to GAPDH and presented as a relative fold change. The information on antibodies is provided in Table S1.

### 5.9 Quantitative PCR (qPCR)

RNA was extracted using the Total RNA Kit (Omega, GA, USA) in accordance with the manufacturer's instructions. Subsequently, RNA was reverse transcribed into cDNA using a reverse transcription kit, followed by Real‐time Quantitative PCR performed with SYBR Green Master Mix (Vazyme, China). GAPDH served as an internal normalization control. All experiments were conducted in triplicate, and the data were analyzed using the 2^−ΔΔCt^ method. The primer sequences are provided in Table S3.

### 5.10 RNA‐Sequencing

pMuSCs were cultured in MDM and CM from pMSCs, respectively, for 3 days. Then, total RNA was extracted from the samples using TRIzol (Sangon Biotech, China). Library preparation and RNA sequencing were conducted by Guangzhou Gene Denovo Biotechnology Co., Ltd. (Guangzhou, China), utilizing three biological replicates per group. Specifically, the mRNA was subsequently enriched and reverse transcribed to generate double‐stranded cDNA. Following end repair, PCR amplification was performed to create a sequencing library. The Agilent 2100 Bioanalyzer was also used for precise evaluation of RNA integrity. Agarose gel electrophoresis was employed to analyze the RNA integrity of the samples and to check for DNA contamination. The NanoPhotometer spectrophotometer was utilized to assess RNA purity, while the Qubit 2.0 Fluorometer provided accurate quantification of RNA concentration. Quality control on raw reads was performed using fastp, which filtered low‐quality data to obtain clean reads. HISAT2 was employed for reference genome alignment, StringTie was used to reconstruct transcripts, and RSEM was utilized to calculate the expression of all genes in each sample. Gene expression levels were quantified based on FPKM (fragments per kilobase of transcript per million mapped reads) values. DESeq2 was used to conduct inter‐group differential analysis to identify significantly different genes between groups. These differentially expressed genes were subsequently subjected to Gene Ontology (GO) analysis and Kyoto Encyclopedia of Genes and Genomes (KEGG) pathway analysis.

### 5.11 DiI Staining

Cells were washed with PBS and incubated with 10 µM 1,1’‐dioctadecyl‐3,3,3′,3′‐tetramethylindocarbocyanine perchlorate (DiL) cell plasmembrane probe (Beyotime, C1036) for 10 min at room temperature. After that, the cells were washed 3 times with PBS and subjected to the indicated assays. Cells labeled with Dil were detected using fluorescence microscopy at 549 nm.

### 5.12 Fabrication of Starch‐Based Scaffolds

Three types of starch‐based scaffolds were fabricated by the freeze‐drying technique. First, 2 % corn starch (C), sweet potato starch (S), and potato starch (P) were dissolved in distilled water at 65°C and mixed with 2 % sodium alginate (SA) solution. These solutions were poured into molds of 1 × 1 × 0.5 cm^3^ and pre‐frozen overnight at −20°C. Next, a 75 % ethanol aqueous solution of 2 % (w/v) CaCl_2_ was added to the molds for a 24 h crosslinking reaction, followed by rinsing with distilled water. This approach was adopted to facilitate the formation of a finer pore structure and enhance scaffold integrity during the subsequent lyophilization process [47]. The scaffolds were then obtained through freeze‐drying and overnight sterilization with 75 % ethanol.

### 5.13 Scanning Electron Microscopy (SEM) Analysis

The morphology of the starch‐based scaffolds in dry states was examined under a SEM (GeminiSEM300, Zeiss, Germany) after sputter coating a thin layer of gold. The images were analyzed by the ImageJ software (National Institutes of Health, NIH) to calculate the number of pores and pore sizes.

### 5.14 Porosity of Starch‐Based Scaffolds

The porosity of the scaffolds was determined using the ethanol displacement method [48]. Briefly, the scaffolds were first weighed (W1) and then immersed in ethanol and ultrasonicated to allow the ethanol to enter the pores. The scaffolds were then removed and weighed again (W2). The porosity of the scaffold was determined by the weight difference, calculated by the following formula:

(1) Porosity(%)=[(W2−W1)/W2]×100

where W1 represents the weight of the scaffold before ethanol displacement, W2 represents the weight of the scaffold after ethanol displacement.

### 5.15 Swelling Ratio of Starch‐Based Scaffolds

The lyophilized scaffolds were weighed and immersed in DMEM for 12, 24, 48, 72, or 96 h. Then, the scaffolds were removed, wiped with filter paper to remove surface moisture, and weighed again. The swelling ratio was determined by calculating the weight difference of the scaffolds before and after immersion using the following formula:

(2) $$\mathrm{Swelling}\;\mathrm{ratio}\;\left(\%\right)=\left[\;\left(\mathrm{Wt}\;-\;W0\right)/W0\right]\;\times \;100$$

where W_0_ represents the weight of the scaffolds in dry states, W_t_ represents the weight of the scaffolds after immersion.

### 5.16 Degradation Ratio of Starch‐Based Scaffolds

The scaffold samples were immersed in DMEM at 37°C for 1 h, then removed, wiped off the surface liquid with filter paper, and weighed as M_0_. Subsequently, the scaffolds were placed back into DMEM at 37°C for 15 days. Every other day, the scaffolds were removed, wiped off the surface liquid with filter paper, and then weighed as Mn (n = 1, 3, 5, 7, 9, 11, 13, and 15). The degradation ratio (%) was calculated by the following equation:

(3) $$\mathrm{Degradation}\;\mathrm{ratio}\;\left(\%\right)=\left[\;1\;-\;\left(\mathrm{Mn}/M0\right)\right]\;\times \;100\;\%$$

### 5.17 Stress‐Strain Curve of Starch‐Based Scaffold

The scaffold samples were immersed in medium for 24 h, then removed, and the surface liquid was removed with filter paper. Then, the uniaxial compression test was performed using an electronic universal testing machine (CMT6503, Shenzhen SANS, China). Each sample was tested at a loading speed of 10 mm/min, with increasing compression until a 85 % strain level was reached.

### 5.18 Cell Viability

To evaluate the cytotoxicity of the starch‐based scaffolds, cell viability assays were performed using the CCK‐8 method. First, the scaffold was cut into 1 cm^3^ pieces and immersed in 1 mL GM. The scaffold leachate was then collected after incubation at 37°C for 24 h. A cell suspension (1 × 10^5^ cells/mL) was added to a 96‐well plate at a volume of 100 µL per well and incubated with the scaffold leachate at 37°C for 24 h. The cell viability was assessed using a CCK8 detection kit (Vazyme, China). The absorbance was measured at 450 nm using a microplate reader (Biotek, Winooski, VT, USA) to quantify the cell viability.

### 5.19 Cell Seeding on the Starch‐Based Scaffolds and Assessment of Proliferative Viability

Prior to cell seeding, the scaffolds were placed in 6‐well plates and soaked in DMEM for 24 h for swelling. Then, pMuSCs and pMSCs were mixed at a ratio of 7:3 and then seeded onto the scaffolds at a cell density of 0.8 × 10^5^ cells/cm^3^, allowing them to adhere for 4 h before adding sufficient GM. The scaffolds containing cells were cultured in a 37°C incubator with 5 % CO_2_ for 6 days.

The cell proliferation rate was determined and analyzed by the Alarma Blue cell proliferation/cytotoxicity assay kit (KeyGEN BioTECH, Cat# KGA9504) according to the instructions. First, a standard curve was established using a cell mixture consisting of pMuSCs and pMSCs in a 7:3 ratio with a gradient concentration from 0.1 × 10^3^/mL to 1 × 10^5^/mL. Every 24 h during culture, the medium was removed and replaced with the Alma Blue solution for a 4 h incubation. Then, the solution was collected to measure fluorescence intensity using a microplate reader (BioTek, Winooski, VT, USA) at an excitation wavelength of 530 nm and an emission wavelength of 590 nm. The number of cells at different time points was calculated according to the standard curve.

### 5.20 Assessment of Myogenic and Adipogenic Co‐Differentiation on the Starch‐Based Scaffolds

pMuSCs and pMSCs were mixed at a ratio of 7:3 and then seeded onto the scaffolds at a cell density of 2 × 10^5^ cells/cm^3^. The cells were first cultured in GM for 24 h and then transferred to the co‐differentiation medium for 3 days. After that, the scaffolds containing cells were washed with PBS and fixed with 4 % PFA at room temperature for 1 h. Then, they were subjected to immunofluorescence staining to detect myotubes and adipocytes. Images were taken using a laser‐scanning confocal microscope (Nikon A1, Japan).

### 5.21 Preparation of Cell‐Based Hybrid Noodles

The S‐SA scaffolds were fabricated with a mold of 10 × 1 × 0.5 cm. After freeze‐drying and sterilization, the scaffolds were placed onto 10 cm peri dishes and soaked in DMEM for 24 h for swelling. Then, the cell mixture consisting of pMuSCs and pMSCs in a 7:3 ratio was seeded onto the scaffolds at a cell density of 2 × 10^6^ cells/cm^3^. The cells were first cultured in GM for 1 day and then transferred to the co‐differentiation medium for 3 days. Then, the scaffolds containing cells were removed and washed with water for 3 times. Subsequently, the scaffolds containing cells were placed in a ventilated area at room temperature to dry for 24 h to obtain cell‐based hybrid noodles.

### 5.22 Nutritional Evaluation of Cell‐Based Hybrid Noodles

The protein content of the cell‐based noodle was determined using the UV absorption method, specifically based on the tyrosine (C_Tyr_) content. Initially, the sample was soaked in a 2 m NaOH solution, with its weight recorded as W, and subsequently heated in a constant temperature water bath at 100°C for 6 h. Once the sample was completely hydrolyzed and cooled to room temperature, it was diluted with deionized water and filtered using filter paper. The filtrate was then further diluted with a 0.1 m NaOH solution, and the absorbance (A_240_) was measured at a wavelength of 240 nm using a microplate reader (Biotek, Winooski, VT, USA). The formula for calculating the protein content in the sample is:

(4) $$\mathrm{Protein}\;\mathrm{content}\;\left(g/100\;g\right)=\left(A240/\left(W\times \mathrm{CTyr}\right)\right)\times 15.123$$

The TG content of the cell‐based noodle was determined using the TG assay kit (Pulilai, China) according to the instructions. First, a standard curve was established using gradient dilution of the standard solution. Then, the cell‐based noodle samples were added to the lysis solution and homogenized. After allowing the samples to stand for 10 min to ensure complete lysis, the working solution was added and mixed thoroughly. Following a 15 min incubation at 37°C, the absorbance was measured at a wavelength of 550 nm using a microplate reader (Biotek, Winooski, VT, USA), and TG content was calculated based on the standard curve. The fat content of the cell‐based noodle was determined by the Soxhlet extraction method following the Chinese national standard “National Food Safety Standard‐Determination of fat in Food” (GB 5009.6–2016). The ash content of the cell‐based noodle was determined by the high‐temperature burning method following the Chinese national standard “National Food Safety Standard‐Determination of ash in Food” (GB 5009.4–2016). The carbohydrate content of the cell‐based noodle was calculated by subtracting the content of the protein, ash, and fat from the total mass.

### 5.23 Amino Acid Profile

The amino acid profile was determined according to the Chinese national standard “National Food Safety Standard‐Determination of Amino Acids in Food” (GB 5009.124–2016). The protein in the sample was hydrolyzed in 6 m hydrochloric acid at a temperature of 120°C for 22 h to convert all amino acids into free amino acids. After hydrolysis, the solution was neutralized with NaOH, filtered through double‐layer filter paper, and centrifuged at 1000 rpm for 10 min to collect the supernatant. Then, the amino acid content of the samples was detected using a reversed‐phase high‐performance liquid chromatography (HPLC) system equipped with a UV detector (Agilent Technologies, CA, USA).

### 5.24 Fatty Acid Profile

The amino acid profile was determined according to the Chinese national standard “National Food Safety Standard‐Determination of Fatty Acids in Food” (GB 5009.168–2016). Briefly, the samples were hydrolyzed with hydrochloric acid, and fat was extracted using an ether‐petroleum ether solution. The ether layer was collected, concentrated to dryness via rotary evaporation, and subjected to fat saponification and fatty acid methyl esterification. Fatty acid analysis was performed using a gas chromatograph with a hydrogen flame ionization detector (Agilent Technologies, CA, USA). The fatty acid content in the samples was quantified using mixed and individual fatty acid methyl ester standards.

### 5.25 Texture Analysis

The cell‐based noodle samples were boiled for in water 5 min, then removed and wiped off the surface water with filter paper. Then, the texture of the cooked noodle samples was analyzed using a texture analyzer (TA Instruments, USA) equipped with a cylindrical 36 mm‐diameter stainless steel plunger (P/36R). The samples were trimmed into 1 × 1 × 0.5 cm^3^ and then compressed twice with the following experimental conditions: a trigger force of 5 g, a compression ratio of 75 %, a pre‐test speed of 2 mm/s, a test speed of 1 mm/s, and a post‐test speed of 2 mm/s. Maintain an interval of 5 s between two compressions. The TPA parameters were calculated using the User Guide Software (Version 1.0).

### 5.26 Water Absorption of Cell‐Based Hybrid Noodles

The cell‐based hybrid noodle in its dry state was weighed and then boiled in water for 5 min. Next, the noodle was removed, wiped with filter paper to remove surface moisture, and weighed again. The water absorption rate was calculated using the formula:

(5) $$\mathrm{Water}\;\mathrm{absorption}\;\mathrm{rate}\;\left(\%\right)=\left[\;\left(\mathrm{Gt}\;-\;G0\right)/G0\right]\;\times \;100$$

where G_0_ represents the weight of the cell‐based hybrid noodle in its dry state, G_t_ represents the weight of the cell‐based hybrid noodle after boiling.

### 5.27 Flavor Analysis

The volatile compounds were first extracted using headspace‐solid phase microextraction (HS‐SPME) and analyzed using a gas chromatograph (GC, Agilent Technologies, CA, USA). First, 1 g of the cell‐based noodle sample was placed in a headspace vial with purified water and incubated at 40°C for 30 min under agitation at 250 rpm. To prevent the compound from leaking, each vial was sealed with parafilm before heating. Then, a solid‐phase microextraction (SPME) fiber was inserted into the headspace vial and maintained at 150°C for 10 min, enabling volatile and semi‐volatile compounds in the sample to be adsorbed onto the fiber. The SPME fiber was then immediately inserted into the GC injection port at 250°C for 8 min to desorb the volatiles. A 60 m × 0.25 mm HP‐INNOWAX capillary column with a 0.25 µm film thickness (J&W Scientific, Folsom, CA, USA) was used to separate the volatile compounds under a 1 mL/min flow rate of helium (carrier gas). The types and content of compounds were identified by comparing the data searched in the spectral library (Agilent Chemstation Integrator) based on the retention time of the standards. The specific flavor note was assigned to each detected volatile compound using the FlavorDB database.

### 5.28 Statistical Analysis

Each experiment was conducted at least three times independently. Data visualization was performed using GraphPad Prism 8 (GraphPad Software Inc., CA, USA). Comparisons between two groups were analyzed using unpaired Student's *t*‐tests, while differences among three or more groups were evaluated using one‐way ANOVA followed by Tukey multiple comparison test. Significant differences are indicated by either asterisks or letters. ^*^ *p* < 0.05, ^**^ *p* < 0.01, and ^***^ *p* < 0.001. The same letters indicate no significant difference (*p* > 0.05), whereas different letters indicate a significant difference (*p* < 0.05).

## Author Contributions

X.G., Z.M., and J.Z. conceptualized and designed the experiments. L.W., W.S., Y.F., and H.T. performed the experiments. X.G., L.W., D.W., and Z.M. analyzed the data. G.D., J.C., and J.Z. supervised the project. X.G., L.W., Z.M., and J.Z. wrote and revised the manuscript. All the authors read and approved the final manuscript.

## Conflicts of Interest

The authors declare no conflict of interest.

## Supporting information




**Supporting File**: advs73740‐sup‐0001‐SuppMat.pdf.

## Data Availability

The data that support the findings of this study are available from the corresponding author upon reasonable request.
