# Supplementary material for: Creating Cell‐Based Hybrid Noodles for Sustainable and Nutrient‐Balanced Diets via a Serum‐Free and Animal‐Free 3D Co‐Differentiation System
Source: Adv Sci (Weinh). 2026 Jan 12;13(16):e19916. doi: 10.1002/advs.202519916 (PMC13042633; doi:10.1002/advs.202519916)
Supplement: Supplementary file 1 — Supporting File: advs73740‐sup‐0001‐SuppMat.pdf. [file ADVS-13-e19916-s001.pdf]

Supporting Information

Creating cell-based hybrid noodles for sustainable and nutrient-balanced diets via a serum-free and animal-free 3D co-differentiation system

Xin Guan [1,2,*], Luyi Wang [1], Wanqiang Sun [1], Yiwei Feng [1], Dandan Wang [1], Haohao Tang [1], Zhenwu Ma [3,*], Guocheng Du [1,2], Jian Chen [1,2] and Jingwen Zhou [1,2,*]

[1] School of Biotechnology and Key Laboratory of Industrial Biotechnology of Ministry of Education, Jiangnan University, Wuxi, China

[2] State Key Laboratory of Food Science and Resources, Jiangnan University, Wuxi, China

[3] College of Mechanical Engineering, Suzhou University of Science and Technology, Suzhou, China

*Corresponding authors

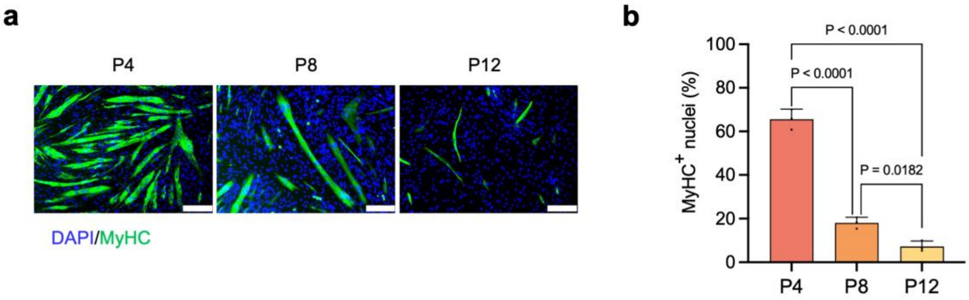

Figure S1. Assessment of the myogenic potential of pMuSCs during *in vitro* expansion. (a) Representative MyHC immunofluorescence staining images after myogenic differentiation of pMuSCs at different passages. Scale bar: 200 μm. (b) Quantification of the percentage of MyHC$^+$ nuclei after myogenic differentiation of pMuSCs of different passages (n = 3 independent experiments). For (a), similar results were obtained in three independent experiments. For (b), error bars indicate means ± SD. Significance was determined by one-way ANOVA with Tukey's post hoc analysis. *p*-values are annotated in the figures.

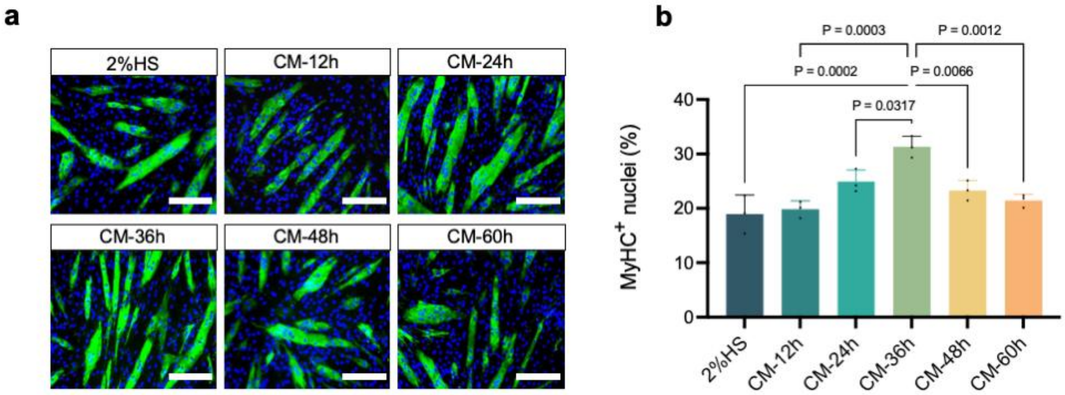

Figure S2. CM from pMSCs enhanced the myogenic efficiency of pMuSCs. (a) Representative MyHC immunofluorescence staining images after culturing pMuSCs with CM from pMSCs at different time points. Scale bar: 200 μm. (b) Quantification of the percentage of MyHC$^+$ nuclei after culturing pMuSCs with CM from pMSCs at different time points (n = 3 independent experiments). For (a), similar results were obtained in three independent experiments. For (b), error bars indicate means ± SD. Significance was determined by one-way ANOVA with Tukey's post hoc analysis. *p*-values are annotated in the figures.

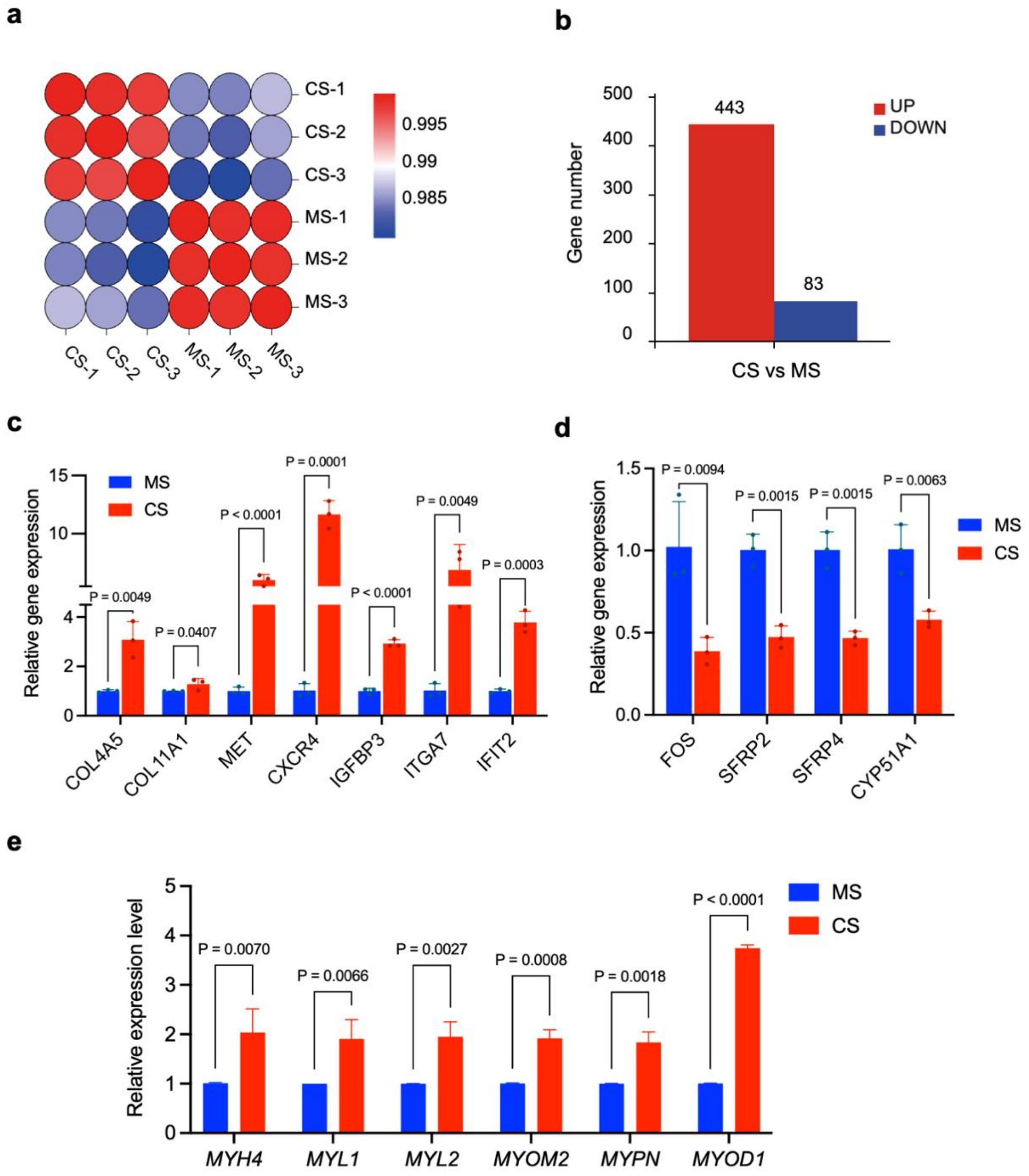

Figure S3. Analysis of transcriptome data between pMuSCs-CS and pMuSCs-MS and validation of representative genes using qPCR. (a) Heat map illustrating the correlation between pMuSCs-CS and pMuSCs-MS samples. (b) Count of differential genes between pMuSCs-CS and pMuSCs-MS, with up-regulated genes shown in red and down-regulated genes in blue. (c) qPCR analysis of representative up-regulated genes involving the ECM remodeling, cell migration, and cell surface receptors. (d) qPCR analysis of representative down-regulated genes involving cell proliferation and Wnt signaling. (e) qPCR analysis of representative genes involving myogenesis. n = 3 independent experiments, error bars indicate means ± SD. For (c–e), significance was determined by Student's *t*-test. *p*-values are annotated in the figures.

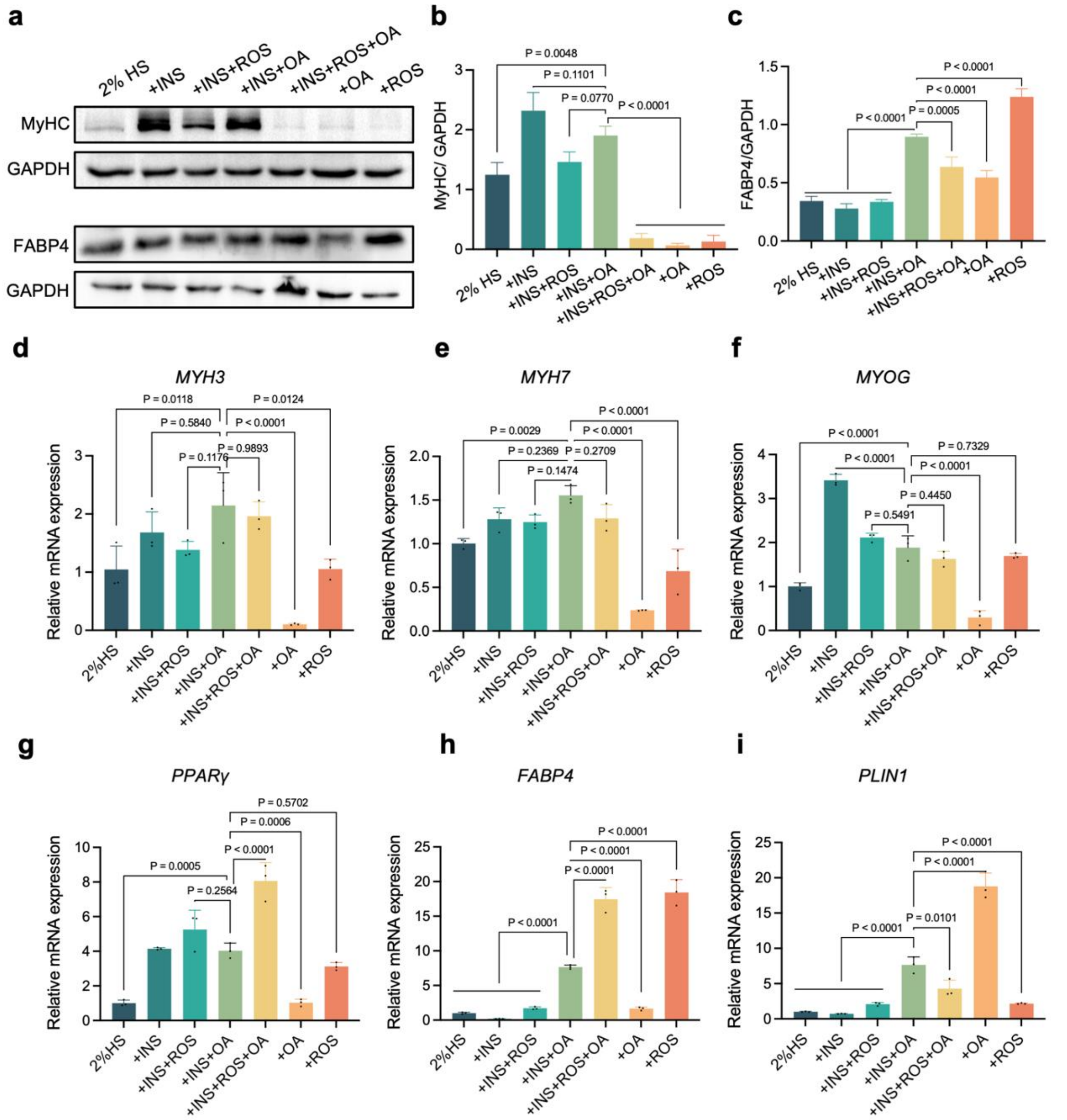

Figure S4. Assessment of protein and mRNA expression in cells after 3 days of treatment with different inducers in the co-culture system. (a) Representative blots of MyHC, FABP4, and GAPDH proteins from cells in the co-culture system following 3 days of treatment with different inducers. (b) Quantification of MyHC expression normalized to GAPDH (n = 3 independent experiments). (c) Quantification of FABP4 expression normalized to GAPDH (n = 3 independent experiments). (d–f) The mRNA expression of genes related to myogenic differentiation (*MYH3*, *MYH7*, and *MYOG*) was quantified by qPCR (n = 3 independent experiments). (g–i) The mRNA expression of genes related to adipogenic differentiation (*PPARγ*, *FABP4*, and *PLIN1*) was quantified by qPCR (n = 3 independent experiments). For (a), similar results were obtained in three independent experiments. For (b–i), error bars indicate means ± SD. Significance was determined by one-way ANOVA with Tukey's post hoc analysis. *p*-values are annotated in the figures.

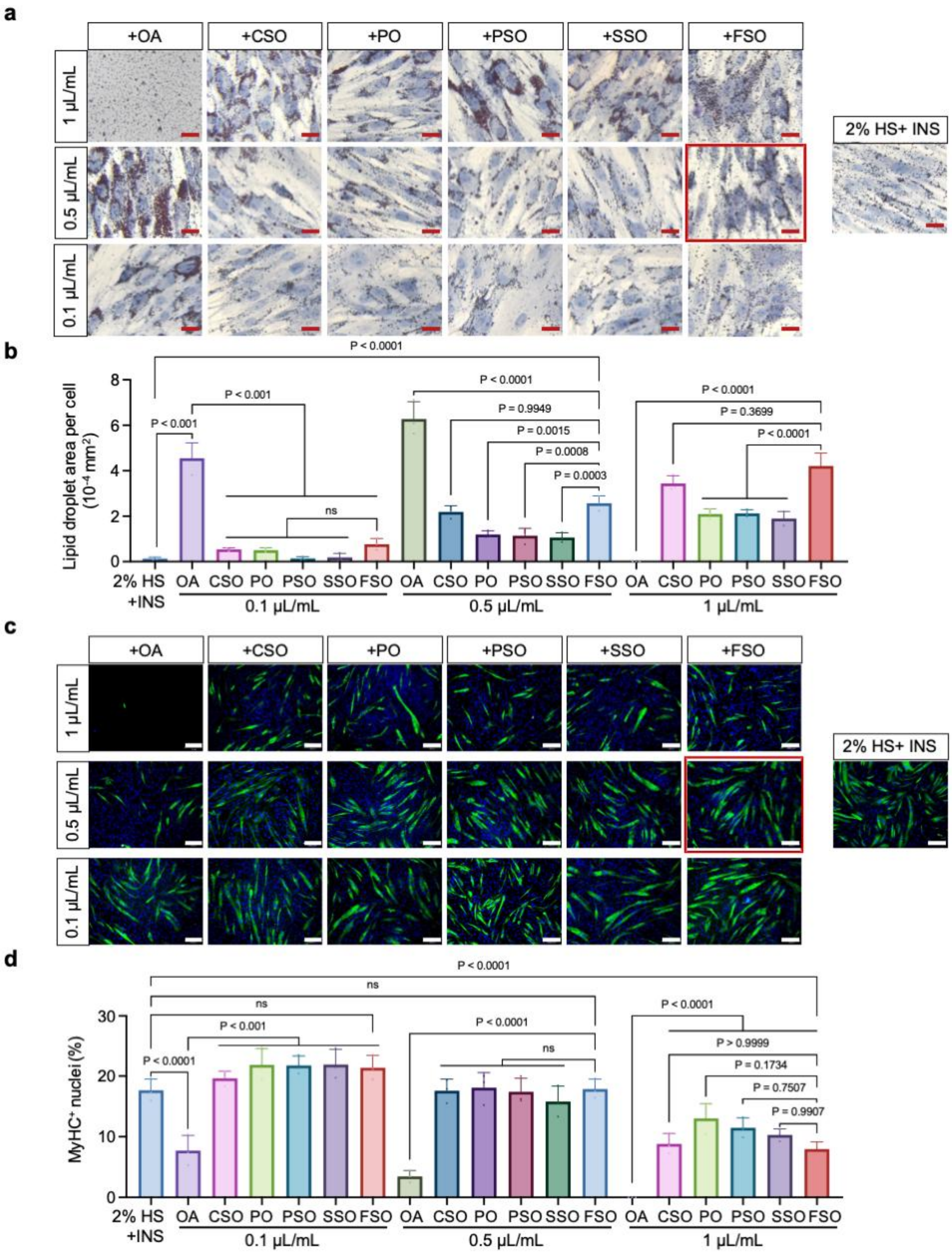

Figure S5. The effect of various edible oils on myogenesis and adipogenesis in the co-culture system. (a) Representative Oil Red O staining images of the co-culture system after 3 days of culture with insulin in combination with either OA or various edible oils. Scale bar: 50 μm. (b) Quantification of lipid droplet area per cell (n = 3 independent experiments). (c) Representative MyHC fluorescence staining images of the co-culture system after 3 days of culture with insulin in combination with either OA or various edible oils. Scale bar: 200 μm. (d) Quantification of the percentage of $MyHC^+$ nuclei (n = 3 independent experiments). For (b, d), error bars indicate means ± SD. Significance was determined by one-way ANOVA with Tukey's post hoc analysis.

p-values are annotated in the figures. For (a, c), similar results were obtained in three independent experiments.

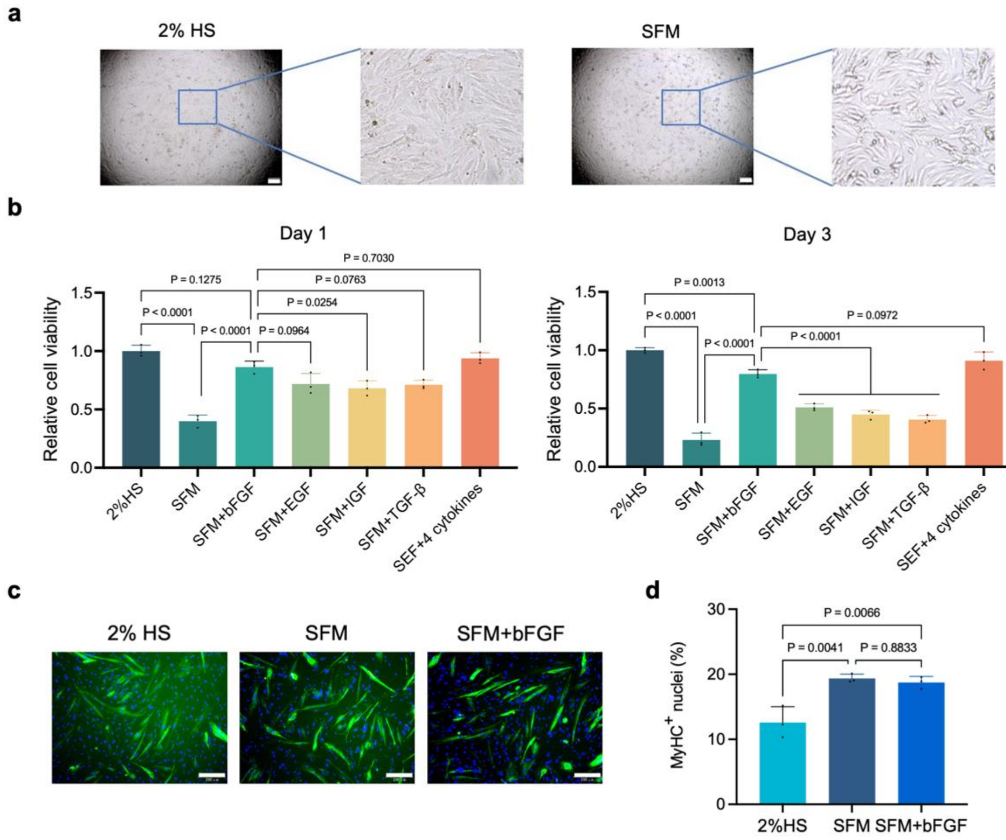

Figure S6. Optimization of SFM to support the cell viability and differentiation in the co-culture system. (a) Representative cell morphology of pMSCs after 24 h of culture in 2% HS and SFM. Scale bar: 200 μm. (b) Relative cell viability of pMSCs at day 1 and day 3 in 2% HS or SFM supplemented with various growth factors (n = 3 independent experiments). (c) Representative MyHC fluorescence staining images of differentiated pMuSCs after 3 days of culture in 2% HS, SFM, or SFM+bFGF. Scale bar: 200 μm. (d) Quantification of the percentage of MyHC^{+} nuclei (n = 3 independent experiments). For (b, d), error bars indicate means ± SD. Significance was determined by one-way ANOVA with Tukey's post hoc analysis. *p*-values are annotated in the figures. For (a, c), similar results were obtained in three independent experiments.

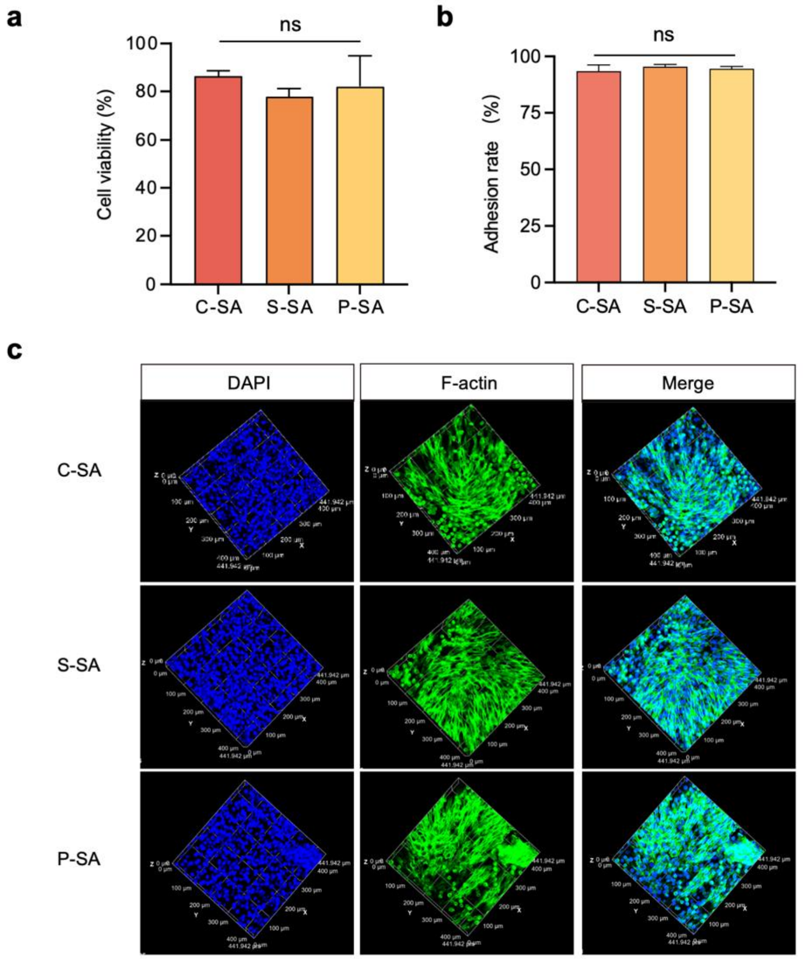

Figure S7. Examination of the cytocompatibility of starch-based scaffolds. (a) The cell viability of co-cultured pMuSCs and pMSCs after 24 h of incubation in the leaching solution of C-SA, S-SA, or P-SA scaffolds (n = 3 independent experiments). (b) The adhesion rate of co-cultured pMuSCs and pMSCs on C-SA, S-SA, or P-SA scaffolds (n = 3 independent experiments). (c) Representative fluorescent phalloidin staining images of co-cultured pMuSCs and pMSCs after 6 days of culture on C-SA, S-SA, or P-SA scaffolds. DAPI was used for nuclear staining. For (a–b), error bars indicate means ± SD. Significance was determined by one-way ANOVA with Tukey's post hoc analysis. ns indicates no significant difference (p >0.05). For (c), similar results were obtained in three independent experiments.

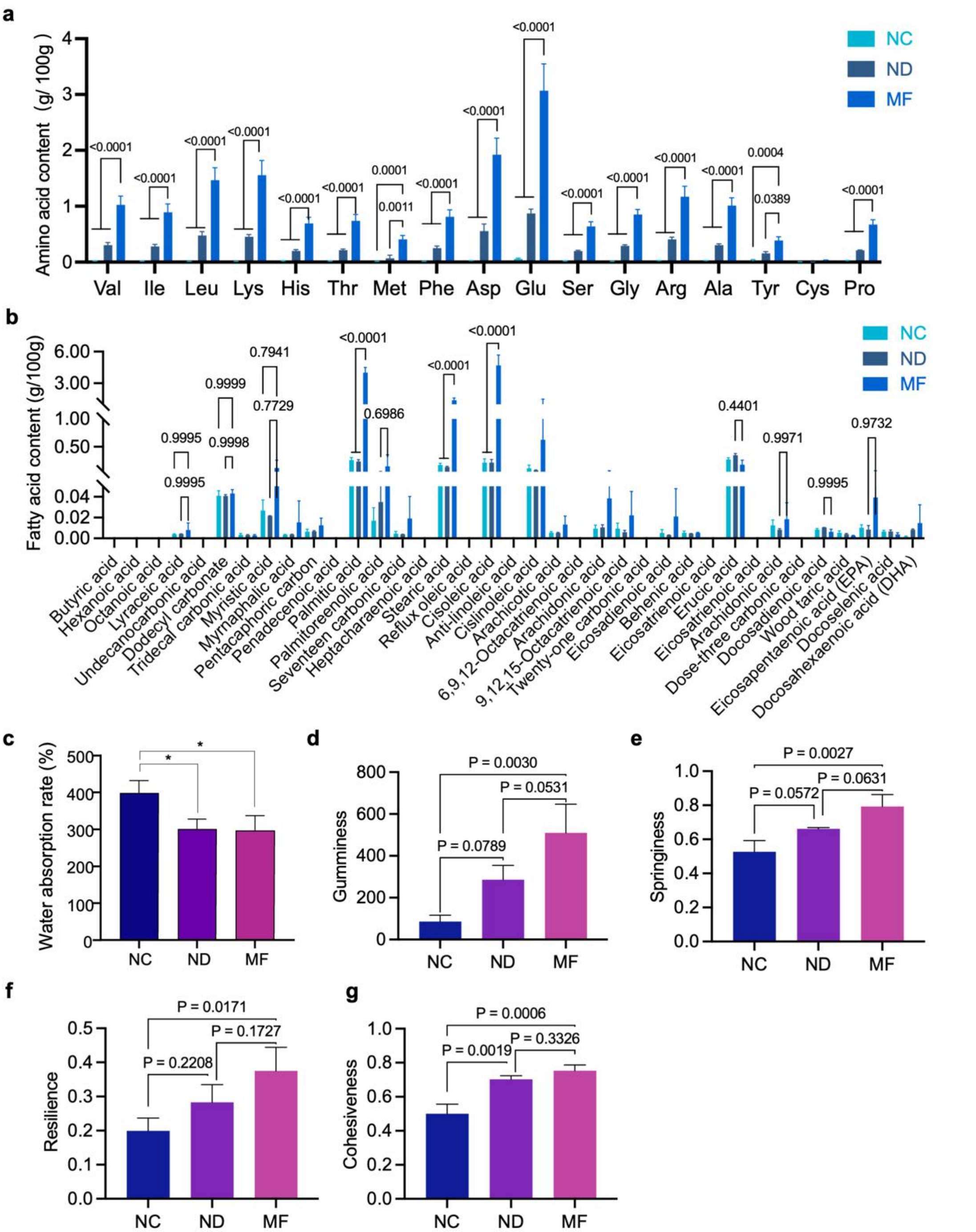

Figure S8. Nutritional and texture assessment of the cell-based hybrid noodle. (a) Amino acid profile of NC, ND, and MF (n = 3 independent experiments). (b) Fatty acid profile of NC, ND, and MF (n = 3 independent experiments). (c) The water absorption rate of NC, ND, and MF after standard cooking (n = 3 independent experiments). (d) Gumminess of NC, ND, and MF (n = 3 independent experiments). (e) Springiness of NC, ND, and MF (n = 3 independent experiments). (f) Resilience of NC, ND, and MF (n = 3 independent experiments). (g) Cohesiveness of NC, ND, and MF (n = 3 independent experiments). For (a–g), error bars indicate means ± SD. Significance was determined by one-way ANOVA with Tukey's post hoc analysis. *p*-values are annotated in the figures.

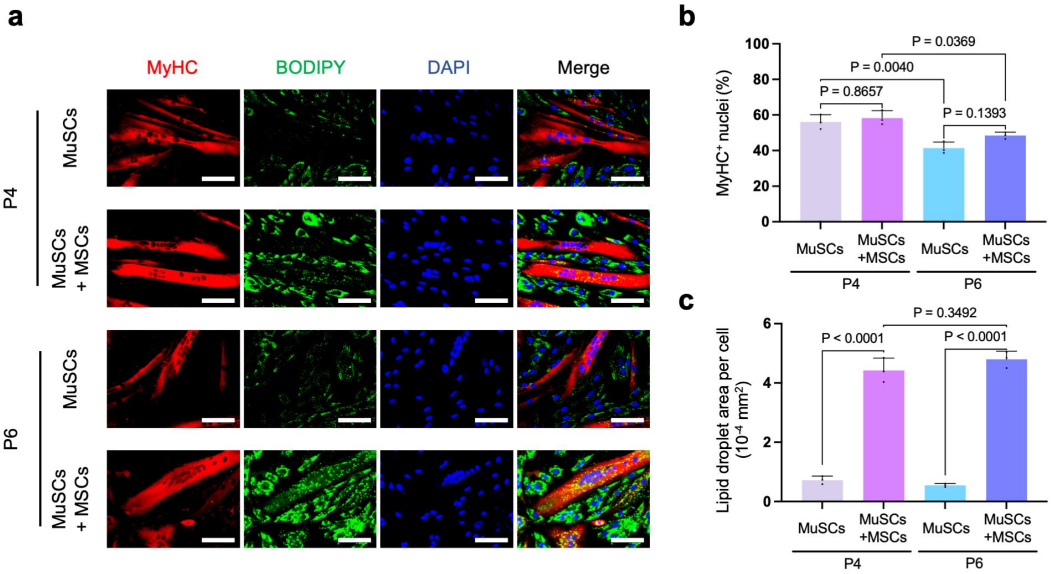

Figure S9. Validation of the co-differentiation medium in early-passage cells. (a) Representative MyHC (red) and Bodipy (green) fluorescence staining images of the co-culture system after 3 days of differentiation induction in early-passage cells (P4 and P6). DAPI was used for nuclear staining (blue). Scale bar: 100 μm. (b) Quantification of the percentage of MyHC$^+$ nuclei (n = 3 independent experiments). (c) Quantification of lipid droplet area (n = 3 independent experiments). For (a), similar results were obtained in three independent experiments. For (b–c), error bars indicate means ± SD. Significance was determined by one-way ANOVA with Tukey's post hoc analysis. *p*-values are annotated in the figures.

Supplementary Tables

Table S1. Key antibodies, chemicals, and recombinant proteins used in this work

**Antibodies**	**Catalog number**	**Dilution ratio**
Mouse monoclonal anti-MYH3	Santa Cruz Biotechnology, Cat# sc-376157	1:1000
Rabbit monoclonal anti-FABP4	MedChemExpress, Cat# HY-P80124	1:1000
GAPDH Monoclonal antibody	Proteintech Group, Cat# 60004-1	1:1000
HRP-conjugated goat anti-rabbit IgG	Proteintech, Cat# SA00001-2	1:1000
HRP-conjugated goat anti-mouse IgG	Proteintech, Cat# SA00001-1	1:1000
DAPI	Sigma-Aldrich, Cat# MBD0015	1:1000
BODIPY 493/503	Thermo Fisher Scientific, Cat# D3922	1:500
CoraLite488-conjugated Goat Anti-Mouse IgG (H+L)	Proteintech, Cat# SA00013-1	1:1000
CoraLite594-conjugated Goat Anti-Mouse lgG(H+L)	Proteintech, Cat# SA00013-3	1:1000
CoraLite® Plus 488-conjugated Phalloidin	Proteintech, Cat# PF00001	1:1000
**Chemicals and Recombinant proteins**	**Catalog number**	
Recombinant human bFGF	GenScript, Cat# Z03116	
Recombinant human EGF	GenScript, Cat# Z02691	
Recombinant human IGF-1	GenScript, Cat# Z03177	
Recombinant human TGF-β	GenScript, Cat# Z03411	
Sodium alginate	Sigma-Aldrich, Cat# W201502	
Corn starch	Sichuan Youjia Foodstuffs	
Sweet potato starch	Sichuan Youjia Foodstuffs	
Potato starch	Sichuan Youjia Foodstuffs	
Trypsin-EDTA	Gibco, Cat# 25300120	
Fetal bovine serum	Gibco, Cat# 16000-044	
Horse serum	Gibco, Cat# 26050088	
DMEM, high glucose, no glutamine	Gibco, Cat# 11960-044	
Insulin	Solarbio, Cat# I8040	
Rosiglitazone	MedChemExpress, Cat# HY-17386	
Oleic acid	Sigma-Aldrich, Cat# O1008	

Table S2. Formulation of SFM

Component	Source	Concentration
DMEM	11965092, ThermoFisher	
BSA	A8010-5, Solarbio	0.5 mg/mL
MEM amino acid solution	11130077, ThermoFisher	0.5%
ITS	41400045, ThermoFisher	0.2%

Table S3. Primer sequences used in this study

Gene	Primer sequences（5′ >3′）
MYH4	F：CCAAATACGAGACGGACGC
	R：CCACATCAAGCATGAGGTCC
MYH2	F：GCAACGCAGAAGAAAAGGC
	F：CATCCAGACGGTGCTGTAGG
MYH3	F：GGAGGGCATCCGCATCTG
	R：CACGCCTTCTTGCTGTCG
MYH7	F：TGAAGGTGGGCAACGAATAC
	R：TGTCCAGGACCCCTATGAAGTA
GAPDH	F：GGGCTGCTTTTAACTCTGGC
	R：TGGGTGGAATCATACTGGAACA
PPARγ	F：TGGCAAAGCACTTGTATGACTC
	R：GCGAAACTGACACCCCTGA
PLIN1	F：CCCTGGTGGCGTCTGTATG
	R：CAGAGCAGCACCGAGGACTT
FABP4	F：GTGGGAGTGGGCTTTGCC
	R：ATGGTGCTCTTGACTTTCCTGT
MYOG	F：CCAGGAACCCCACTTCTATGA
	R：GGTCCCCAGCCCCTTATC
MYL1	F：GGTGATGTCCTTCGGGCTCT
	R：GCTTCCCTGGTCCTTGTTGTT
MYL2	F：TGCTCAGGGCTGATTATGTAAA
	R：TCTCCGTGGGTGATGATGTG
MYOM2	F：GTCGCCGTGCCCTTCTAC
	R：TCTGGCTGGACGCCCTCT
MYPN	F：TCGCTTGGAACGTACCCC

	R：CGGAAGTGCTTGAGTCTTTTGT
Desmin	F：AGCCGAAGAGTGGTACAAGTCA
	R：CCTCATCAGGGAATCGTTAGTG
COL4A5	F: AAGGGTGAACCAGGCTTTCA
	R: CCTGGCAGACCTGGAGGA
COL11A1	F: CTCTGAAGGTCCCCAGGGT
	R: AATACCAGGAGCGCCGTTG
MET	F: TCTGCCGTCTGTGCATTCC
	R: TGATGAATTTCTCAAAAGTGTCCTG
CXCR4	F: TGGACGGGTTCCGTATATTCA
	R: CCCGGAAACAGGGTTCCTTT
IGFBP3	F: ACAAGAAAAAGCAGTGCCGC
	R: CCGTACTTATCCACGCACCA
ITGA7	F: AGTGGACATTGACCGTGGTG
	R: TCGATGCGCACAGGTAACAA
FOS	F: GACACACTCCAAGCGGAGAC
	R: TCATCAGGGATCTTGCAGGC
SFRP2	F: TATGTCCGCGTTCGGCTTC
	R: TTCACATACCTTTGGAGCTTCCT
SFRP4	F: CCCTCGAACTCAAGTCCCAC
	R: AGAAGCATCATTCGTGAGCG
